# MMP8 exacerbates sepsis-induced pulmonary vascular leakage by disruption of endothelial VE-cadherin through ERK signalling

**DOI:** 10.1186/s43556-026-00522-4

**Published:** 2026-07-24

**Authors:** Yaojun Peng, Qiyan Wu, Yuyu Liu, Di Jing, Yang Bai, Haiyan Zhu

**Affiliations:** 1 Department of Emergency, The First Medical Center of Chinese, PLA General Hospital, Beijing, China; 2 Medical School of Chinese PLA, Beijing, China; 3 Laboratory of Oncology, The First Medical Center of Chinese, PLA General Hospital, Beijing, China; 4 Institute of Oncology, The Fifth Medical Center of Chinese, PLA General Hospital, Beijing, China

**Keywords:** MMP8, Sepsis, Vascular leakage, VE-cadherin, Endocytosis, Endothelial cells

## Abstract

Endothelial dysfunction is a major contributor to multi-organ failure and mortality in sepsis, with the lungs being particularly susceptible to vascular leakage. Previous studies have implicated matrix metalloproteinase 8 (MMP8) in sepsis pathogenesis. However, the role of MMP8 in maintaining endothelial barrier integrity during sepsis remains unclear. This study aimed to investigate the role of MMP8 in sepsis-induced vascular leakage and the underlying mechanisms. A significant increase in MMP8 expression was observed in lipopolysaccharide (LPS)-treated endothelial cells and lung tissues from mice with cecum ligation and puncture-induced sepsis. In vitro, loss- or gain-of-function of MMP8 in endothelial cells modulated LPS- or Cytomix (TNF-α, IL-1β and IFN-γ)-induced endothelial hyperpermeability, without inducing cell apoptosis or affecting cell viability. In vivo, pharmacologic inhibition of MMP8 alleviated pulmonary vascular leakage, multi-organ injury, and mortality in septic mice. Mechanistically, MMP8 interacted with ERK, promoting its phosphorylation, and consequently activated calpains, leading to membrane VE-cadherin proteolysis. This induced VE-cadherin internalisation through clathrin- and caveolin 1-mediated endocytosis. Clinically, serum MMP8 levels were elevated in patients with sepsis, with the highest levels observed in those with pleural effusion. A nomogram incorporating albumin, blood urea nitrogen, lactate, Acute Physiology and Chronic Health Evaluation II score, and serum MMP8 levels showed favourable prediction accuracy and clinical utility for pleural effusion risk. These findings suggest the critical role of MMP8 in sepsis and its potential as a promising biomarker of sepsis-induced pulmonary vascular leakage.

**Supplementary Information:**

The online version contains supplementary material available at 10.1186/s43556-026-00522-4.

## Introduction

Sepsis is a life-threatening syndrome characterised by multi-organ dysfunction resulting from a dysregulated host response to infection [1]. Among affected organs, the lung is particularly susceptible to injury during sepsis [2]. Sepsis‑induced acute lung injury (ALI), with acute respiratory distress syndrome (ARDS) representing its most severe form, is driven by complex inflammatory cascades involving multiple cell populations, including endothelial cells (ECs) and inflammatory phagocytes [3].

Recent studies have shown that sepsis‑induced ALI is commonly associated with increased vascular leakage and pulmonary oedema [4, 5]. Endothelial permeability is governed by homotypic interactions of VE‑cadherin (CDH5), an essential constituent of endothelial adherens junctions that preserve endothelial junctional integrity and maintain pulmonary fluid homeostasis [6, 7]. Transcriptional modulation of *VE‑cadherin*, together with endocytosis and proteolytic degradation of VE‑cadherin mediated by phosphorylation‑ and ubiquitination‑dependent signalling cascades, contributes to augmented endothelial permeability [8]. This ultimately disrupts structure and function of the adherens junctions, representing a major driver of sepsis-induced pulmonary vascular leakage [9]. Uncovering the diverse regulatory signals targeting VE‑cadherin may help design novel therapeutic targets for sepsis intervention.

Matrix metalloproteinase 8 (MMP8), also referred to as neutrophil collagenase, belongs to the MMP family with more than 20 members and plays important roles in degradation and remodelling of the extracellular matrix (ECM) [10]. MMP8 is expressed in a variety of cell types, including neutrophils, ECs, smooth muscle cells, fibroblasts, dendritic cells, and macrophages [11]. Emerging evidence indicates that MMP8 is dysregulated in sepsis and regulates the progression of sepsis through multiple cell‑specific mechanisms. Maiorov et al. demonstrated that MMP8, released from activated neutrophils and macrophages, was markedly elevated in sepsis, and targeted collagen I of the cardiac ECM to induces diastolic dysfunction [12]. Lu et al. found that MMP8 was significantly increased in septic dendritic cells, where it regulated dendritic cell tolerance in late polymicrobial sepsis via the NF-κB p65/β-catenin pathway [13]. However, the role of MMP8 in ECs under septic condition remains poorly understood, and its effect on endothelial permeability has not been fully elucidated.

This study aimed to investigate the role of MMP8 in sepsis-induced pulmonary vascular leakage and the underlying molecular mechanisms. Our hypothesis is that MMP8 regulates VE-cadherin disruption in ECs to promote sepsis-induced vascular leakage. Loss- or gain-of-function analysis of MMP8 function in vitro was conducted using lentiviral transduction in ECs. In vivo pharmacologic inhibition of MMP8 was carried out in mice with sepsis induced by caecal ligation and puncture (CLP). Upregulation of MMP8 was found in ECs upon septic challenge, and ERK signalling was involved in MMP8-mediated endocytosis of VE-cadherin. A nomogram with serum MMP8 level included was developed to predict the risk of sepsis-induced pleural effusion. These findings suggest that MMP8 plays a critical role in sepsis and may serve as a promising biomarker of sepsis-induced pulmonary vascular leakage.

## Results

### MMP8 expression is markedly elevated in ECs upon septic challenge

**Fig. 1:**
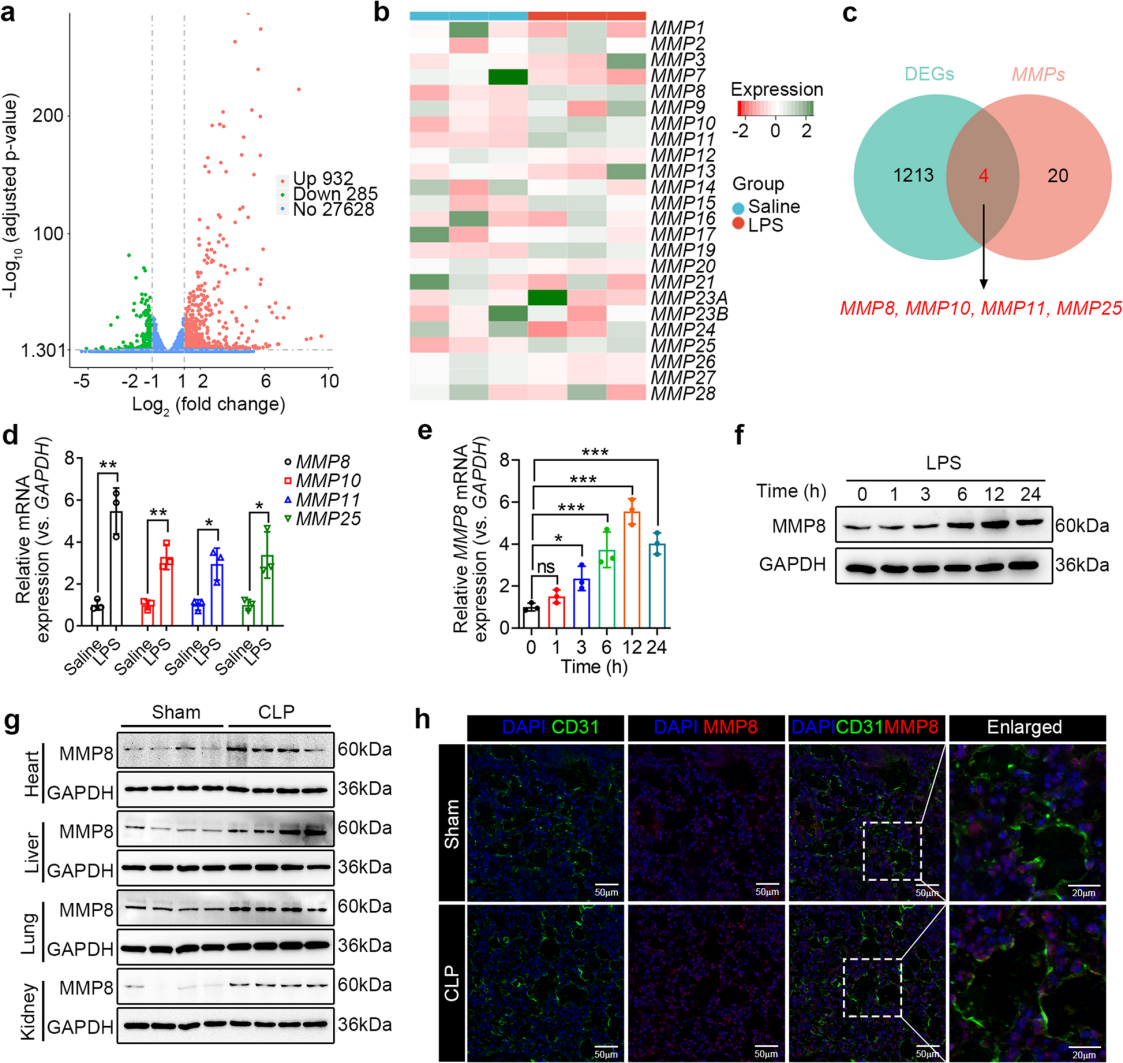
Expression pattern of MMPs in ECs upon septic challenge. **a** DEGs between saline- and LPS-treated HUVECs measured by RNA-seq. The screen criteria were | log_2_ (fold change) |> 1 and adjusted *p* < 0.05. **b** Expression profile of MMPs in HUVECs treated with LPS or saline. **c** Venn diagram showing differentially expressed MMPs. **d** qPCR analysis of mRNA expression of *MMP8*, *MMP10*, *MMP11*, and *MMP25* in HUVECs treated with LPS or saline (*n* = 3 per group). **e** Time-dependent expression of *MMP8* mRNA in HUVECs upon LPS challenge (*n* = 3 per group). **f** Representative immunoblots showing time-dependent expression of MMP8 protein in HUVECs upon LPS challenge. **g** Representative immunoblots of MMP8 in the heat, liver, lung and kidney of sham or CLP mice. **h** Representative immunofluorescence images showing the expression of MMP8 (red) and its colocalization with CD31 (green), an endothelial marker, in the lung of sham or CLP mice; scale bar: 50 μm for the left three columns, 20 μm for the right column. Data are shown as mean ± SD; p values were calculated using unpaired Student’s t test (**d**) and one-way ANOVA with Dunnett’s t test (**e**); ∗ *p* < 0.05, ∗ ∗ *p* < 0.01, and ∗ ∗ ∗ *p* < 0.001. ANOVA, Analysis of variance; CLP, Cecum ligation and puncture; DEGs, Differentially expressed genes; ECs, Endothelial cells; LPS, Lipopolysaccharide; MMPs, Matrix metalloproteinases

To evaluate the expression of various MMPs in ECs during sepsis, HUVECs were treated with saline or LPS (10 μg/mL) for 12 h and harvested for RNA-seq. Differential expression analysis revealed 932 upregulated and 285 downregulated differentially expressed genes (DEGs) according to the screening criteria (| log_2_ (fold-change) |> 1 and adjusted *p* < 0.05) (Fig. 1a). An heatmap showed the expression of *MMP* family members in HUVECs (Fig. 1b). Intersection analysis of DEGs and MMPs revealed four MMPs, including *MMP8*, *MMP10*, *MMP11* and *MMP25*, which were significantly dysregulated in HUVECs upon LPS stimulation (Fig. 1c). *MMP8*, *MMP10*, *MMP11* and *MMP25* expression increased in LPS-treated HUVECs, with *MMP8* showing the greatest increase (approximately 12-fold) (Fig. 1b). The transcriptional expression of the four *MMPs* was confirmed by qPCR, and *MMP8* consistently exhibited the highest change in expression (Fig. 1d). Therefore, we focused on MMP8 in subsequent analyses.

Dynamic expression of MMP8 was further examined at transcription and protein levels. mRNA levels of *MMP8* in HUVECs upon LPS stimulation reached a peak 12 h post-treatment (Fig. 1e). Western blot analysis revealed a similar expression pattern for MMP8 at the protein level in HUVECs upon LPS stimulation (Fig. 1f).

Next, MMP8 expression was examined in vivo using a model of CLP-induced sepsis in mice. Western blot analysis revealed that MMP8 expression levels were markedly increased in multiple organs (heart, liver, lung, and kidney) of CLP-treated mice compared to sham control mice (Fig. 1g). Immunofluorescence assays showed that MMP8 and CD31—an endothelial marker—colocalised in the lungs, and the fluorescent intensity of MMP8 was greatly enhanced in CLP-treated mice (Fig. 1h). Collectively, these data suggest that MMP8 expression is significantly upregulated in ECs following septic stimulation.

### Loss- or gain-of-function of MMP8 in ECs modulates sepsis-induced endothelial hyperpermeability

**Fig. 2:**
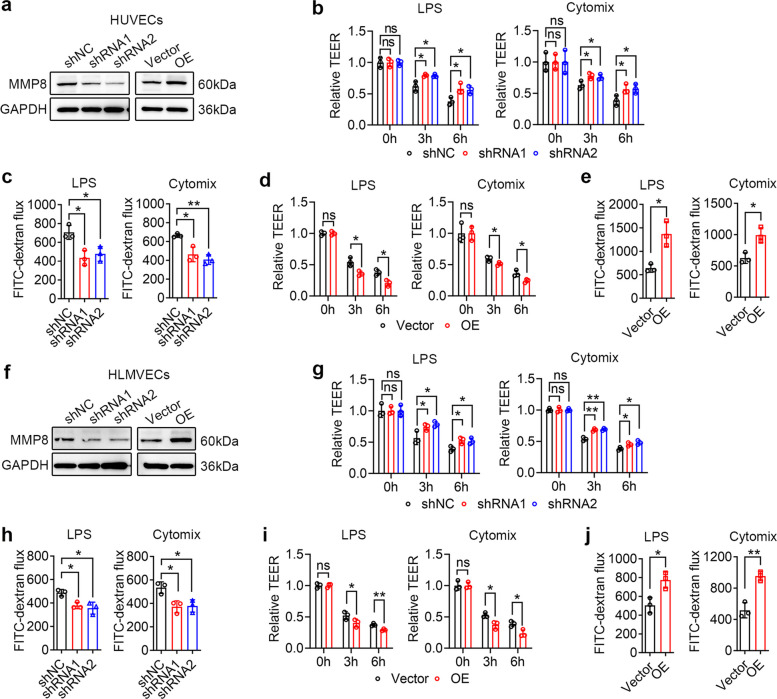
Knockdown or overexpression of *MMP8* in ECs modulates vascular barrier integrity in vitro.** a** Western blot detecting MMP8 knockdown or overexpression in HUVECs. Endothelial permeability was assessed by TEER measurement at 0, 3, 6 h post LPS or Cytomix treatment and FITC-dextran flux at 6 h post LPS or Cytomix treatment in *MMP8*-knockdown (**b**, **c**) or overexpressed (**d**, **e**) HUVECs. **f** Western blot detecting MMP8 knockdown or overexpression in HLMVECs (*n* = 3 per group). Endothelial permeability was assessed by TEER measurement and FITC-dextran flux in *MMP8*-knockdown (**g**, **h**) or overexpressed (**i**, **j**) HLMVECs (*n* = 3 per group). Data are shown as mean ± SD; p values were calculated using one-way ANOVA with Dunnett’s t test (**b**, **c**, **g**, **h**) and unpaired Student’s t test (**d**, **e**, **i**, **j**); ∗ *p* < 0.05. ANOVA, Analysis of variance; ECs, Endothelial cells; LPS, Lipopolysaccharide; MMP8, Matrix metalloproteinase 8

Short hairpin RNA-mediated knockdown or overexpression of *MMP8* was performed in HUVECs using lentiviral transduction. Gene transfer efficiency was examined by checking GFP fluorescence intensity at 48 h post-transduction (Fig. S1a). Knockdown or overexpression of *MMP8* in HUVECs was confirmed by semi-quantitative PCR (Fig. S1b) and Western blot (Fig. 2a).

The effects of MMP8 on the apoptosis and viability of ECs under basal or septic conditions were evaluated using flow cytometry and cell counting kit-8 (CCK-8) assay, respectively. In vitro septic conditions were induced via LPS or Cytomix treatment. No significant changes in cell apoptosis and cell viability were caused by *MMP8* knockdown in HUVECs under LPS or Cytomix stimulation (Fig. S2a-c). Next, trans-endothelial electrical resistance (TEER) and FITC-dextran flux assays were performed to examine the impact of MMP8 on endothelial permeability. In TEER assay, no difference in basal barrier function was observed between *MMP8*-knockdown and control HUVECs (Fig. 2b). However, the LPS- or Cytomix- induced reduction in TEER values at 3 and 6 h post-treatment was lower in *MMP8*-knockdown HUVECs than in control cells (Fig. 2b). Consistently, fluxes of fluorescein isothiocyanate (FITC)-dextran at 6 h post-treatment were significantly lower in *MMP8*-knockdown HUVECs than in control cells (Fig. 2c). Conversely, *MMP8* gain-of-function exacerbated LPS- or Cytomix-induced endothelial hyperpermeability (Fig. 2d, e) but had no effect on cell apoptosis or cell viability (Fig. S2d-f).

Next, human lung microvascular endothelial cells (HLMVECs) were introduced to examine possible heterogeneity in endothelial cell responses. *MMP8* knockdown or overexpression conditions were successfully established in HLMVECs (Fig. 2f). TEER and FITC-dextran flux assays demonstrated that *MMP8* knockdown mitigated LPS- or Cytomix-induced endothelial hyperpermeability (Fig. 2g, h), while *MMP8* overexpression aggravated this phenotype in HLMVECs (Fig. 2i, j). The results were consistent with the findings in HUVECs. Taken together, above findings suggest that MMP8 is involved in sepsis-induced endothelial hyperpermeability.

### MMP8 regulates ERK signalling and VE-cadherin disruption in ECs

**Fig. 3:**
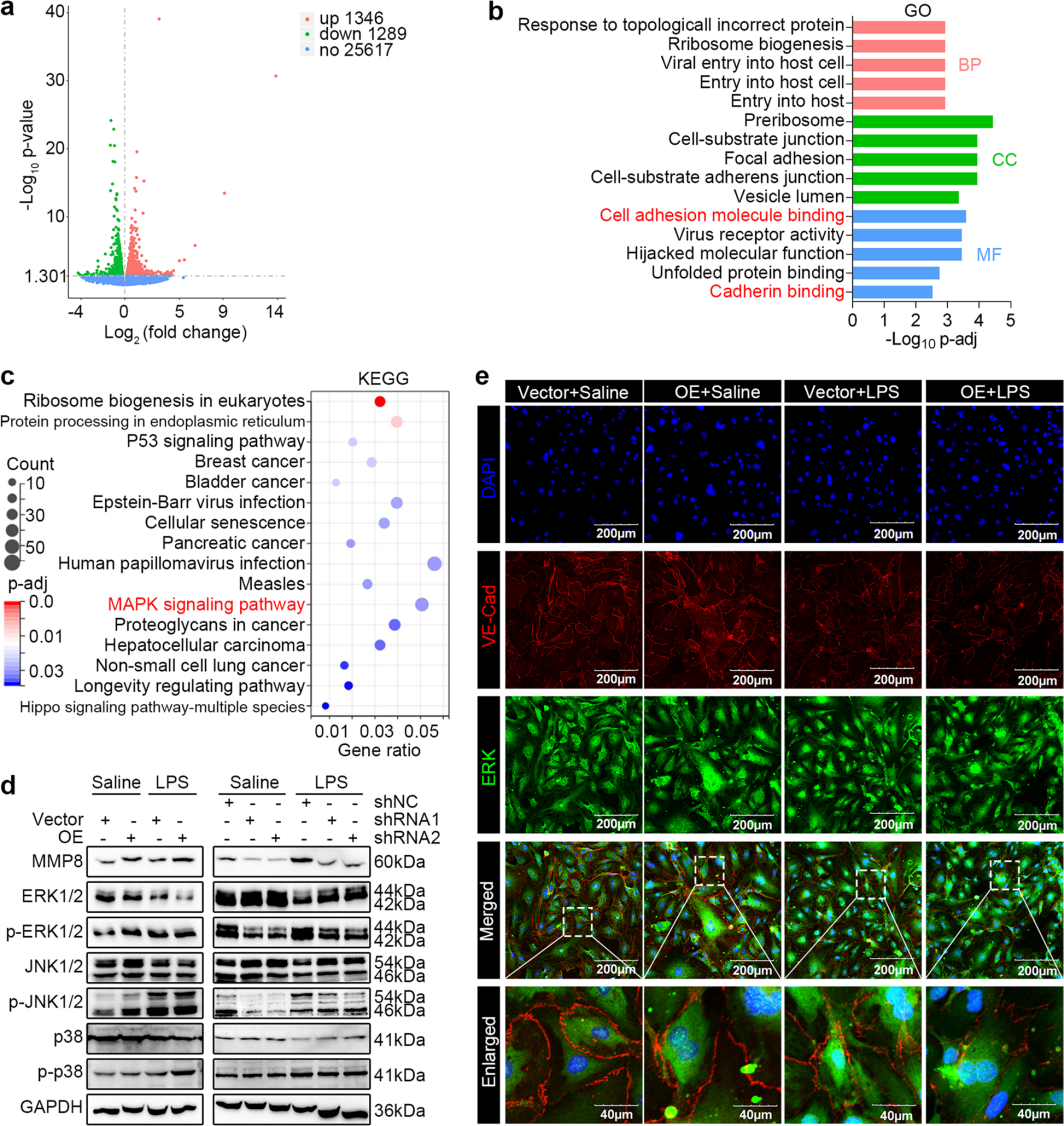
MMP8 regulates ERK signalling and VE-cadherin disruption in ECs.** a** Volcano plot showing differentially expressed genes in *MMP8*-overexpressed HUVECs measured by RNA-seq. The screen criteria were | log_2_ (fold change) |> 0 and *p* < 0.05.** b** Bar plot showing the top 15 significantly enriched GO pathways.** c** Bubble chart showing the top 15 significantly enriched KEGG pathways. **d** Representative immunoblots showing ERK1/2, p-ERK1/2, JNK, p-JNK, p38, and p-p38 expression in *MMP8*-overexpressed or knockdown HUVECs. **e** Representative immunofluorescence images showing VE-cadherin (red) expression and its colocalization with ERK1/2 (green) in *MMP8*-overexpressed or control HUVECs; scale bar: 200 for the top four rows, 40 μm for the last row. ECs, endothelial cells; GO, gene ontology; KEEG, Kyoto Encyclopedia of Genes and Genome; MMP8, matrix metalloproteinase 8; VE-Cad, VE-cadherin

To dissect the mechanism underlying MMP8-regulated endothelial permeability, we performed RNA-seq to profile gene expression in MMP8-overexpressing and control HUVECs. A total of 2,635 DEGs were identified according to the screening criteria (| log_2_ (fold change) |> 0 and *p* < 0.05) (Fig. 3a). Gene ontology (GO) analysis indicated that these DEGs were mainly involved in signalling pathways related to cell junctions and cell adhesion, including cell adhesion molecule binding and cadherin binding (Fig. 3b). Kyoto Encyclopedia of Genes and Genomes (KEGG) analysis suggested that these DEGs were enriched in multiple functional signalling pathways, including MAPK, cellular senescence, and Hippo pathways (Fig. 3c).

VE-cadherin is a key protein present in adherens junctions, and disruption of the VE-cadherin complex impairs endothelial junction formation and vascular integrity [14]. MAPK signalling plays a crucial role in sepsis [15], and the MAPK marker gene ERK is required to maintain endothelial integrity [16]. A recent study has indicated that ERK phosphorylation is an important step in disrupting VE-cadherin integrity [17]. Based on the pathway enrichment results and previous findings, we speculated that MMP8 may regulate ERK signalling-mediated VE-cadherin disruption to promote endothelial permeability.

To test this possibility, the expression and phosphorylation of three players of MAPK (ERK1/2, JNK1/2 and p38) in *MMP8* overexpressed and control HUVECs were examined. *MMP8* overexpression in HUVECs markedly increased the levels of phosphorylated ERK1/2, JNK1/2 and p38 (Fig. 3d). Conversely, *MMP8* knockdown in HUVECs strongly attenuated the phosphorylation of ERK1/2, JNK1/2, and p38 (Fig. 3d). Immunofluorescence assay showed that co-localisation of ERK1/2 and VE-cadherin in HUVECs was more pronounced in confluent than in disperse cells (Fig. S3a), consistent with previous findings [17]. In LPS-treated HUVECs, VE-cadherin expression was markedly decreased in a time-dependent manner (Fig. S3b). Immunofluorescence assay showed that *MMP8* overexpression in HUVECs induced the disassociation of ERK1/2 from VE-cadherin, which was accompanied by more pronounced VE-cadherin disruption (Fig. 3e). Taken together, these results suggest that MMP8 is involved in the regulation of ERK signalling and VE-cadherin disruption in ECs.

### MMP8 interacts with ERK and VE-cadherin in ECs

**Fig. 4:**
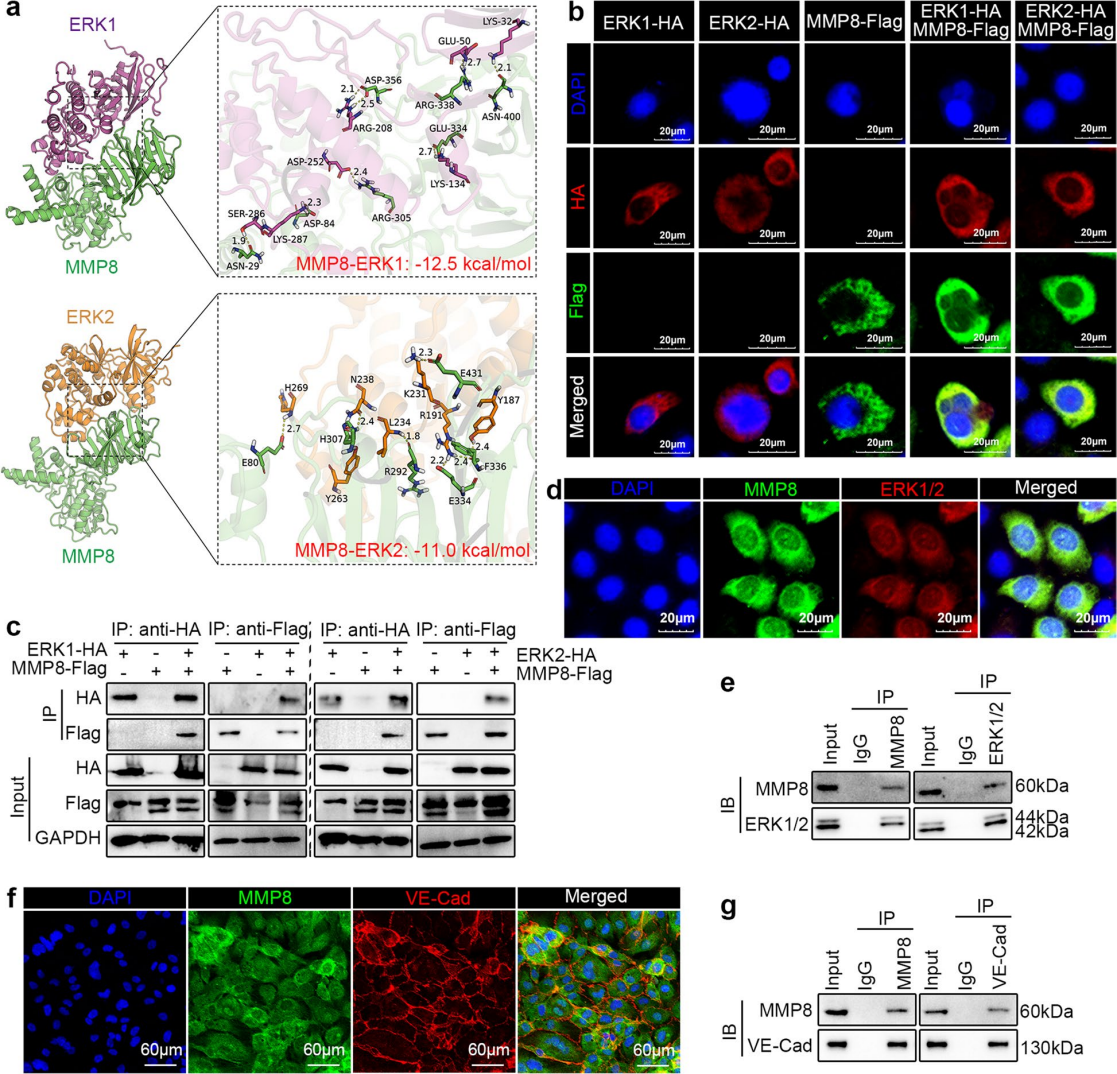
MMP8 interacts with ERK and VE-cadherin in ECs.** a** Three-dimensional simulation diagrams of MMP8 (green)-ERK1 (purple) and MMP8 (green)-ERK2 (amber) by molecular docking. **b** Representative immunofluorescence images showing MMP8-ERK1 and MMP8-ERK2 colocalization in HeLa cells transfected with Flag-tagged MMP8, HA-tagged ERK1, and HA-tagged ERK2; scale bar: 20 μm. **c** Representative immunoblots of Co-IP showing MMP8-ERK1 and MMP8-ERK2 interactions in HeLa cells. **d** Representative immunofluorescence images showing MMP8 (green) and ERK (red) colocalization in HUVECs; scale bar: 20 μm. **e** Representative immunoblots of Co-IP showing the interaction between MMP8 and ERK in HUVECs. **f** Representative immunofluorescence images showing MMP8 (green) and VE-cadherin (red) colocalization in HUVECs; scale bar: 60 μm. **g** Representative immunoblots of Co-IP showing the interaction between MMP8 and VE-cadherin in HUVECs. ECs, endothelial cells; MMP8, matrix metalloproteinase 8; VE-Cad, VE-cadherin

To investigate how MMP8 regulates ERK signalling and VE-cadherin disruption in ECs, molecular docking was used to explore possible interactions between MMP8 and ERK. The three-dimensional structures of the MMP8-ERK1 and MMP8-ERK2 binding complex were predicted, and the predicted binding affinity of MMP8-ERK1 and MMP8-ERK2 were −12.5 and −11.0 kcal/mol, respectively (Fig. 4a). HeLa cells were transiently transfected with plasmids containing Flag-tagged *MMP8*, HA-tagged *ERK1* or HA-tagged *ERK2*, and immunofluorescence assay showed that the green fluorescence-labelled Flag and red fluorescence-labelled HA overlapped, indicating a spatial interaction between MMP8 and ERK (Fig. 4b). Additionally, exogenous co-immunoprecipitation (Co‑IP) experiments in HeLa cells showed the interaction between MMP8 and ERK (Fig. 4c). Furthermore, immunofluorescence assay in HUVECs demonstrated a colocalization of MMP8 and ERK (Fig. 4d). Endogenous Co‑IP assay in HUVECs (Fig. 4e) and mouse lung tissues (Fig. S4a) further indicated the interaction between MMP8 and ERK.

Next, we investigated whether MMP8 interacts with VE-cadherin. The colocalization of MMP8 and VE-cadherin was observed using immunofluorescence assay in HUVECs (Fig. 4f) and mouse lung tissues (Fig. S4b). Co-IP assay in HUVECs further indicated the interaction between MMP8 and VE-cadherin in ECs (Fig. 4g).

### MMP8 promotes VE-cadherin endocytosis through ERK signalling in ECs

**Fig. 5:**
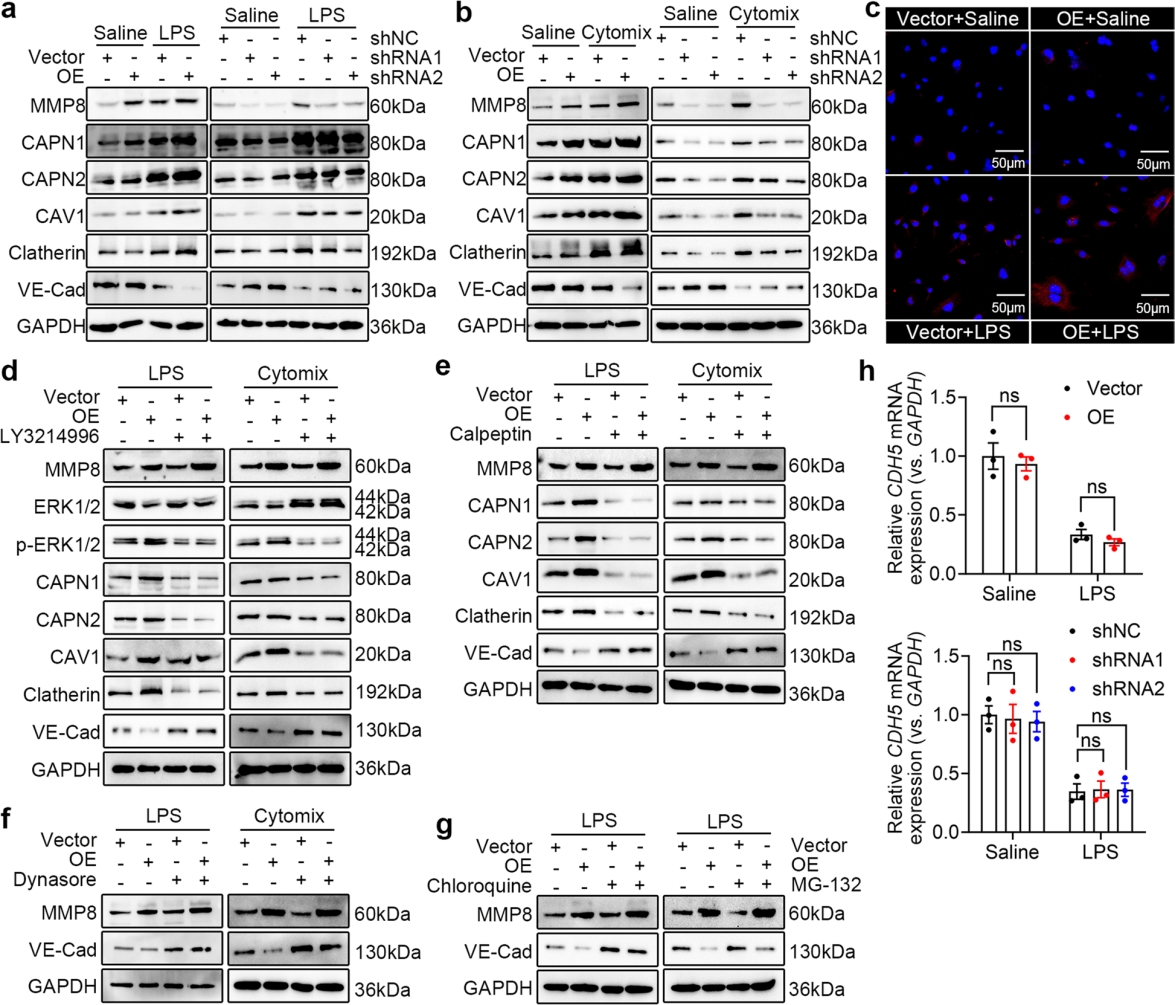
MMP8 promotes VE-cadherin endocytosis through ERK signalling in ECs. Representative immunoblots showing expression of CAPN1, CAPN2, CAV1, clathrin, and VE-cadherin in *MMP8*-overexpressed or knockdown HUVECs treated with LPS or saline (**a**), and in *MMP8*-overexpressed or knockdown HUVECs treated with Cytomix or saline (**b**). **c** Representative immunofluorescence images showing VE-cadherin internalisation (red) in *MMP8*-overexpressed or control HUVECs; scale bar: 50 μm. **d** Representative immunoblots showing expression of ERK1/2, p-ERK1/2, CAPN1, CAPN2, CAV1, clathrin, and VE-cadherin in *MMP8*-overexpressed or control HUVECs treated with an ERK inhibitor (LY3214996).** e** Representative immunoblots showing expression of CAPN1, CAPN2, CAV1, clathrin, and VE-cadherin in *MMP8*-overexpressed or control HUVECs treated with a calpain inhibitor (Calpeptin). **f** Representative immunoblots showing VE-cadherin expression in *MMP8*-overexpressed or control HUVECs treated with a dynamin inhibitor (Dynasore). **g** Representative immunoblots showing VE-cadherin expression in *MMP8*-overexpressed or control HUVECs treated with a lysosomal inhibitor (chloroquine) or a proteasome inhibitor (MG-132). **h** qPCR analysis of *VE-cadherin* in MMP8-overexpressed or knockdown HUVECs. Data are shown as mean ± SD; *p* values were calculated using unpaired Student’s t test (**h**, upper panel) and one-way ANOVA with Dunnett’s t test (**h**, lower panel); ns, no significance. ANOVA, analysis of variance; ECs, endothelial cells; MMP8, matrix metalloproteinase 8; LPS, lipopolysaccharide; VE-Cad, VE-cadherin

Calpains are intracellular Ca^2+^-dependent cysteine proteases that are ubiquitously expressed in mammalian cells [18]. A previous study showed that ERK mediated calpains activation independent of Ca^2+^ to endocytosis [19]. Clathrin and caveolin 1 (CAV1) are key mediators of endocytosis, and previous studies have revealed their critical role in VE-cadherin endocytosis [20–22]. Whether MMP8-mediated ERK phosphorylation activates calpains and the subsequent endocytosis of VE-cadherin in ECs remains unknown. Western blotting showed that calpain 1 (CAPN1) and calpain 2 (CAPN2) were greatly elevated in *MMP8*-overexpressed HUVECs upon LPS or Cytomix stimulation (Fig. 5a, b). Clathrin and CAV1 levels were higher in *MMP8*-overexpressed HUVECs upon LPS or Cytomix stimulation, which was accomplished by lower levels of VE-cadherin (Fig. 5a, b). Conversely, *MMP8* knockdown in HUVECs remarkably attenuated CAPN1/2, clathrin and CAV1 expression upon LPS or Cytomix stimulation, which was accomplished by higher levels of VE-cadherin (Fig. 5a, b). Internalised VE-cadherin was detected by acid washing HUVECs incubated with anti-VE-cadherin antibody and chloroquine. *MMP8* overexpression in HUVECs enhanced LPS-induced increase in VE-cadherin endocytosis (Fig. 5c).

*MMP8*-overexpressed and control HUVECs were treated with an ERK inhibitor (LY3214996) before LPS or Cytomix stimulation. LY3214996 markedly attenuated *MMP8* overexpression-caused increases in CAPN1/2, clathrin and CAV1, accomplished by decrease in VE-cadherin disruption (Fig. 5d), suggesting that endocytosis of VE-cadherin is initiated by MMP8-mediated ERK phosphorylation. *MMP8*-overexpressed and control HUVECs were treated with a calpain inhibitor (calpeptin). Calpeptin markedly attenuated *MMP8* overexpression-induced increase in clathrin and CAV1 and reduced VE-cadherin disruption (Fig. 5e), suggesting that calpains activation is required for the proteolytic cleavage of VE-cadherin. Dynamin is a large GTPase required for clathrin- and CAV1-dependent endocytosis. It forms helical polymers around the necks of budding vesicles and mediates their fission from the plasma membrane [23, 24]. Suppression of clathrin- and CAV1-dependent endocytosis using the dynamin inhibitor (dynasore) prevented *MMP8* overexpression-caused VE-cadherin disruption (Fig. 5f).

Lysosome-dependent degradation has been implicated in the terminal degradation of endocytic VE-cadherin [25]. VE-cadherin can also be targeted by the proteasome for its degradation [26]. We tested whether lysosome or proteasome activity is involved in MMP8-triggered VE-cadherin disruption. *MMP8*-overexpressed and control HUVECs were treated with a lysosomal inhibitor (chloroquine) or a proteasome inhibitor (MG-132). Chloroquine could partially rescue *MMP8* overexpression-induced disruption of VE-cadherin, while MG132 exhibited a weaker rescue effect against VE‑cadherin disruption (Fig. 5g).

In addition, we examined the effect of MMP8 on *VE-cadherin* gene expression. *MMP8* knockdown or overexpression had no influence on *VE-cadherin* transcription in HUVECs (Fig. 5h). Collectively, above results suggest that MMP8 interacts with ERK to promote the endocytosis of VE-cadherin in ECs.

### Pharmacologic inhibition of MMP8 alleviates pulmonary vascular leakage, multi-organ injury, and septic mortality in CLP mice

**Fig. 6:**
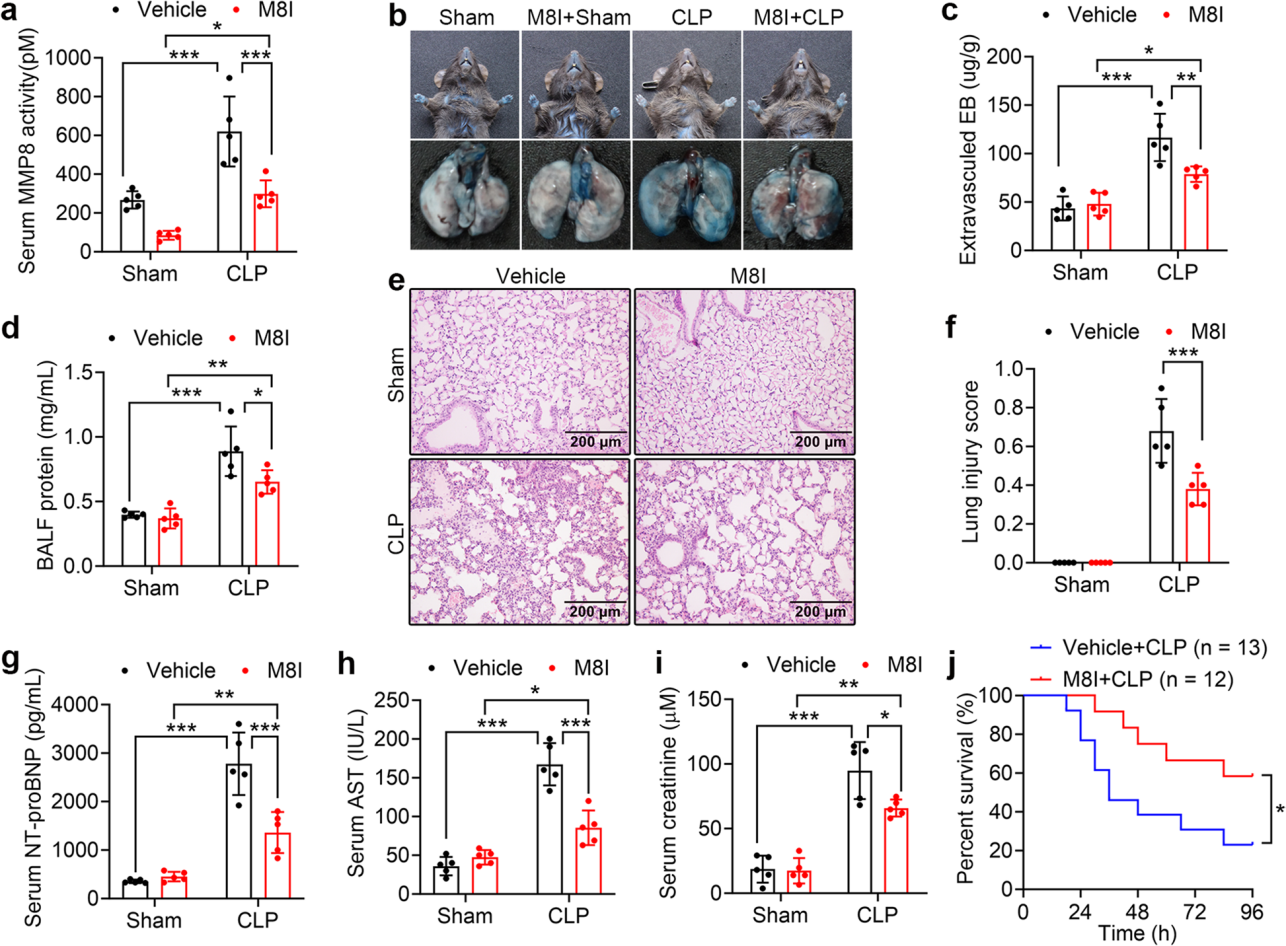
MMP8 inhibition alleviates pulmonary vascular leakage, organ injury, and mortality in septic mice.** a** Measurement of MMP8 enzyme activity in sham-operated or CLP mice (*n* = 5 per group). **b** Representative images showing EB dye circulated in the lip and extremity of MMP8 inhibitor (M8I)- or vehicle-treated mice at 2 h post EB injection (upper panel). Gross images of the lung collected from M8I- or vehicle-treated mice (lower panel). **c** Quantification of EB dye retained within the lung by formamide elution and measurement of absorbance at optical density 620 nm (*n* = 5 per group). **d** BALF was collected at 24 h post CLP or sham operation in M8I- or vehicle-treated mice, and the protein concentration was measured by bicinchoninic acid method (*n* = 5 per group). **e** Representative H&E images of the lung 24 h post CLP or sham operation in M8I- or vehicle-treated mice; scale bar: 200 μm. **f** Quantification of lung injury score (*n* = 5 per group). **g** Assessment of serum NT-proBNP by ELISA. Quantification of serum creatinine concentration (**h**) and AST activity (**i**) by colorimetric assays (*n* = 5 per group). **j** Kaplan–Meier survival curves comparing mortality between M8I- (*n* = 12) or vehicle-treated (*n* = 13) septic mice. Data are shown as mean ± SD; p values were calculated using two-way ANOVA with Tukey’s test (**a**, **c**, **d**, **f**, **g, h**, **i**) and log-rank test (**j**); ∗ *p* < 0.05, ∗ ∗ *p* < 0.01, and ∗ ∗ ∗ *p* < 0.001. ANOVA, analysis of variance; CLP, cecum ligation and puncture; EB, Evans blue; ECs, endothelial cells; MMP8, matrix metalloproteinase 8; BALF, bronchoalveolar lavage fluid

To determine in vivo effects of MMP8 on vascular permeability during sepsis, mice were treated with a MMP8 inhibitor (M8I) or vehicle (1% DMSO) every 12 h beginning 24 h before CLP or sham surgery. Serum MMP8 enzyme activity was significantly elevated in CLP mice compared to sham-operated mice, and M8I treatment significantly inhibited MMP8 enzyme activity compared to the vehicle treatment (Fig. 6a). In vivo vascular permeability was examined using Evans blue (EB) leakage assay. M8I-treated septic mice displayed better peripheral circulation, as manifested by stronger signals in the lips and extremities (Fig. 6b), indicating lower EB dye leakage into internal organs. Next, the lungs of the mice were harvested for visual and quantitative analyses. Weaker blue staining was observed in the lungs of M8I-treated septic mice compared to vehicle-treated septic mice (Fig. 6b). Accordingly, quantification analysis by formamide elution revealed significantly less tissue-extravasated EB dye in the lungs of M8I-treated septic mice (Fig. 6c). Protein concentrations in the bronchoalveolar lavage fluid (BALF) from M8I-treated septic mice were significantly lower than those in vehicle-treated septic mice (Fig. 6d). Histological analysis of the lungs from vehicle-treated mice displayed prominent alveolar collapse, alveolar wall thickening, and dense cellular infiltration, whereas lungs from M8I-treated mice showed milder pathological changes (Fig. 6e). The lung injury scores of M8I-treated septic mice were significantly lower than those of vehicle-treated septic mice (Fig. 6f). Above results suggest that inhibition of MMP8 mitigates pulmonary vascular leakage and lung injury in septic mice.

Given that endothelial hyperpermeability is a key driver of multi-organ dysfunction and even death in sepsis [27], we examined whether MMP8 inhibition had an impact on organ function and survival outcomes during sepsis. Serum N-terminal pro-B-type natriuretic peptide (NT-proBNP), creatinine (Cr), and aspartate aminotransferase (AST) levels were significantly lower in M8I-treated septic mice than in vehicle-treated mice (Fig. 6g-i). Survival analysis showed that M8I-treated septic mice exhibited significantly higher survival rates than vehicle controls at 96 h post-CLP (Fig. 6j). These results suggest that inhibition of MMP8 alleviates injury of multi-organ (heart, renal and liver) and septic mortality in CLP mice.

In addition, we examined the expression of ERK, CAPN1/2, CAV1, clathrin and VE-cadherin in lung tissues of mice, and found that M8I treatment caused decreased ERK phosphorylation and expression levels of CAPN1/2, CAV1 and clathrin, accomplished by increased expression of VE-cadherin (Fig. S4c). These results further imply that MMP8 promotes VE-cadherin endocytosis through ERK signalling.

### MMP8 may be a promising biomarker of pulmonary vascular leakage in patients with sepsis

**Fig. 7:**
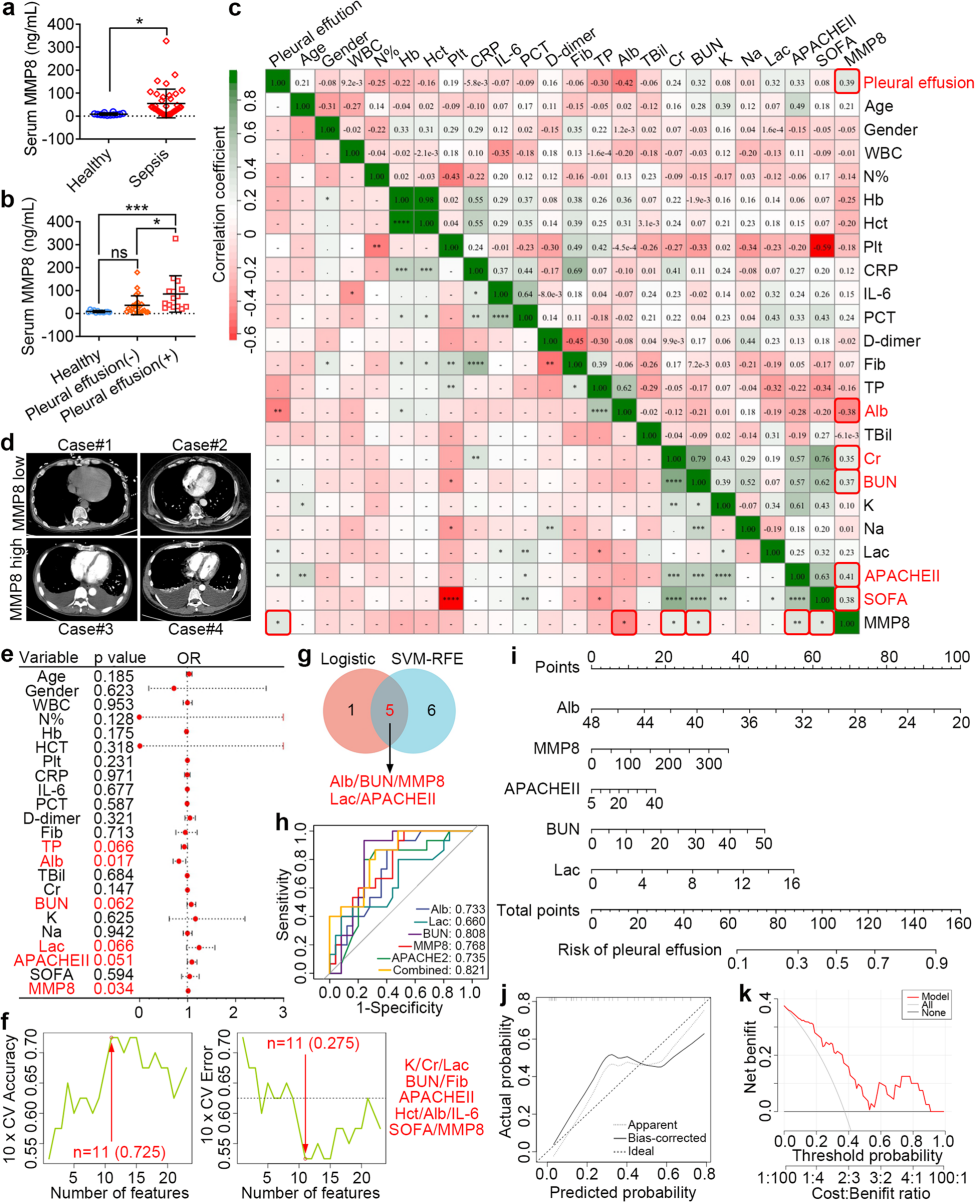
Construction of a nomogram for calculating the risk of pleural effusion in septic patients.** a** Serum MMP8 levels in healthy individuals (*n* = 12) and septic patients (*n* = 40).** b** Serum MMP8 levels in healthy individuals (*n* = 12), septic patients without pleural effusion (*n* = 25), septic patients with pleural effusion (*n* = 15). **c** Heatmap showing Spearman’s correlation coefficients between every two individual clinical parameters of the septic patients. **d** Representative images of chest CT showing pleural effusion in septic patients with differing serum MMP8. **e** Forest plot showing candidate features associated with pleural effusion screened by univariate logistic regression with *p* < 0.1.** f** SVM-RFE determined the optimal threshold at 11 candidate features, achieving the lowest error rate and highest accuracy.** g** Venn diagram showing common candidate features identified by univariate logistic regression and SVM-RFE. **h** ROC curves showing the AUCs of Alb, BUN, Lac, APACHE II score, serum MMP8, and their combination for pleural effusion prediction. **i** A nomogram constructed based on the five common candidate features to calculate risk of pleural effusion. **j** Calibration curve showing the prediction accuracy of the nomogram model. **k** DCA showing the clinical utility of the nomogram model. Data are shown as mean ± SD; p values were calculated using unpaired Student’s t test (**a**) and one-way ANOVA with Tukey’s test (**b**); ∗ *p* < 0.05 and ∗ ∗ ∗ *p* < 0.001; ns, No significance. Alb, Albumin; ANOVA, Analysis of variance; BUN, Blood urea nitrogen; CT, Computed tomography; MMP8, Matrix metalloproteinase 8; SVM-RFE, Support vector machine‑recursive feature elimination; DCA, Decision curve analysis

Finally, we investigated the clinical significance of MMP8 in patients with sepsis. Blood samples were collected from patients with sepsis (*n* = 40) and healthy individuals (*n* = 12). Pleural effusion is a typical clinical manifestation of pulmonary vascular leakage in patients with sepsis. Twenty-five patients with sepsis had pleural effusion, which was diagnosed using chest computed tomography (CT). The demographic, clinical, and laboratory characteristics of the participants are summarised in Table S1. Serum MMP8 levels were significantly higher in patients with sepsis than in healthy individuals (Fig. 7a). Serum MMP8 levels in sepsis patients with pleural effusion were significantly higher than those in patients without pleural effusion. In contrast, the difference in serum MMP8 levels between sepsis patients without pleural effusion and healthy controls was not significant (Fig. 7b). Correlation analysis showed that serum MMP8 was positively correlated with Cr, blood urea nitrogen (BUN), and Sequential Organ Failure Assessment (SOFA) and Acute Physiology and Chronic Health Evaluation II (APACHE II) scores and negatively correlated with albumin (Alb) (Fig. 7c). Septic patients with higher serum MMP8 tended to have more pleural effusion (Fig. 7d).

Next, a nomogram for calculating the risk of sepsis-induced pulmonary vascular leakage was developed. Pleural effusion was set as the dependent variable, and univariate logistic regression and support vector machine‑recursive feature elimination (SVM-RFE) methods were employed to identify candidate features from multiple clinical parameters. Univariate logistic regression analysis identified total protein (TP), Alb, BUN, lactate (Lac), APACHE II score, and serum MMP8 level as candidate features (*p* < 0.1) (Fig. 7e). SVM-RFE analysis identified 11 candidate features, including K, creatinine (Cr), Lac, BUN, fibrinogen (Fib), APACHE II score, haematocrit (Hct), Alb, interleukin-6 (IL-6), SOFA score, and serum MMP8 levels (Fig. 7f). Intersectional analysis yielded a final list of five features: Alb, BUN, Lac, APACHE II score, and serum MMP8 (Fig. 7g). Five common candidate features were merged using multivariate logistic regression. Receiver operating characteristic (ROC) curve analysis was carried out, and the area under curves (AUCs) of Alb, BUN, Lac, APACHE II score, serum MMP8, and their combination for pleural effusion prediction were calculated. The AUC for the combination was the highest at 0.821 (Fig. 7h). Finally, a nomogram based on these five features was constructed (Fig. 7i). The calibration curve analysis showed that the nomogram model had favourable prediction accuracy (Fig. 7j), and the decision curve analysis (DCA) indicated that the nomogram model had good clinical utility (Fig. 7k).

## Discussion

**Fig. 8:**
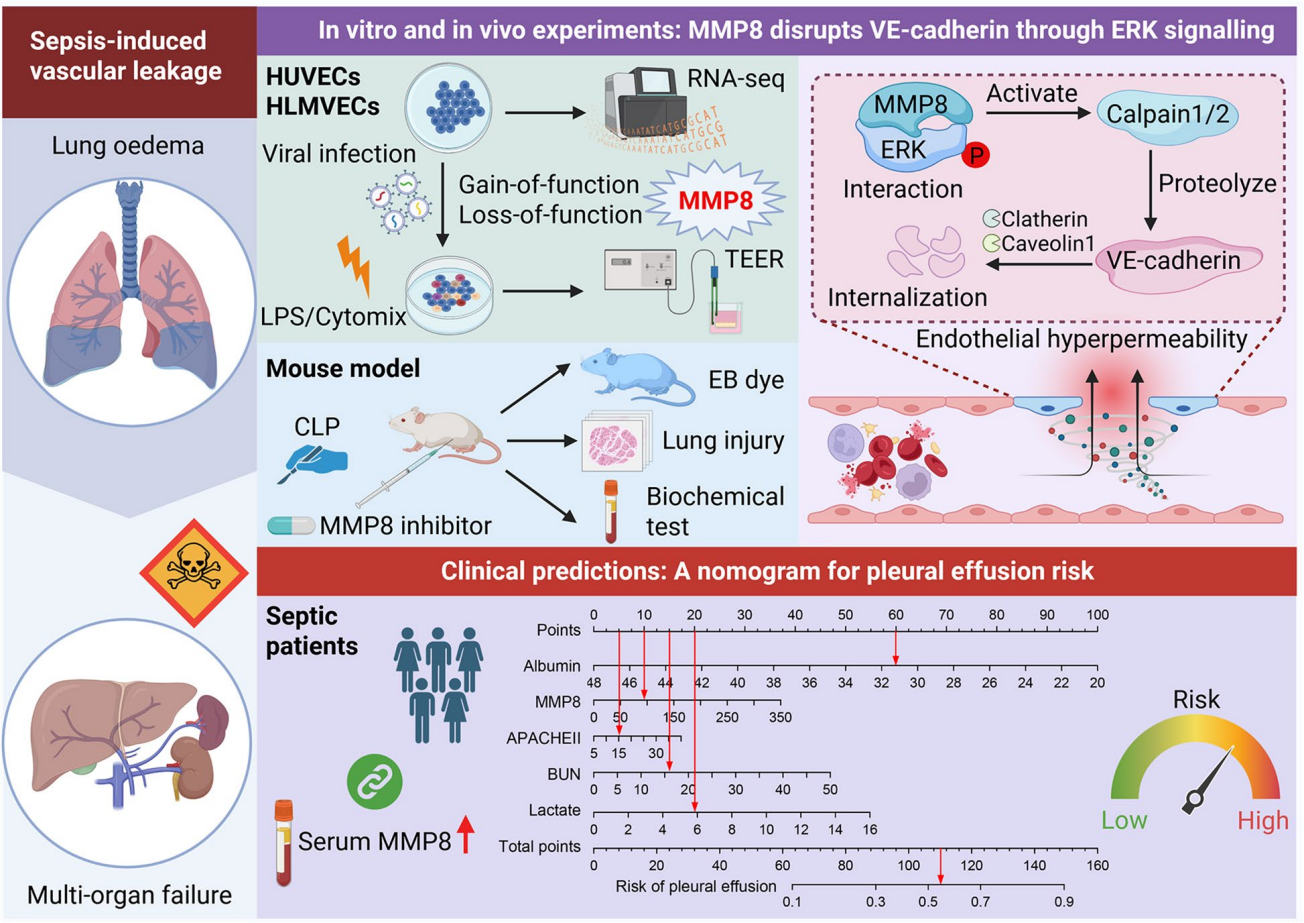
A schematic summary of the study. LPS, lipopolysaccharide; Cytomix, a cocktail of TNF-α, IL-1β and IFN-γ; TEER, trans-endothelial electrical resistance; CLP, caecal ligation and puncture; EB, Evans blue; BUN, blood urea nitrogen; APACHE II, Acute Physiology and Chronic Health Evaluation II; HUVECs, human umbilical vein endothelial cells; HLMVECs, human lung microvascular endothelial cells. Created in BioRender. Peng, Y. (2026) https://BioRender.com/4yvieg0

To our knowledge, this study is the first to demonstrate that sepsis‑induced upregulation of MMP8 in ECs promotes endothelial hyperpermeability. Functionally, endothelial MMP8 promotes in vitro ECs hyperpermeability under septic conditions, and aggravates in vivo vascular leakage, multi-organ injury, and mouse mortality following septic challenge. Mechanistically, MMP8 interacts with ERK, promotes ERK phosphorylation, and consequently activates CANP1/2, leading to VE-cadherin proteolysis on the endothelial membrane. Clathrin- and CAV1-mediated endocytosis might be involved in VE-cadherin internalisation. Clinically, serum MMP8 levels are elevated in patients with sepsis, with the highest levels observed in those with pleural effusion. A nomogram incorporating Alb, BUN, Lac, APACHE II score, and serum MMP8 levels demonstrated favourable prediction accuracy and clinical utility for pleural effusion risk. These findings identify a previously unrecognised MMP8-ERK-calpains-VE-cadherin axis in septic endothelial dysfunction, suggesting the critical role of MMP8 in sepsis and its potential as a promising biomarker of sepsis-induced pulmonary vascular leakage (Fig. 8).

MMP8, also known as neutrophil collagenase or collagenase-2, was initially thought to be expressed solely in mature neutrophils and functionally restricted to ECM degradation [28]. Recent findings suggest that MMP8 is widely expressed in various cell types where it is involved in the pathogenesis of sepsis. Circulating MMP8 levels have been shown to be 35-fold higher in patients with sepsis than in healthy individuals, significantly exceeding the increases observed in other MMPs and their tissue inhibitors [29]. Elevated MMP8 expression correlates with sepsis severity, increased organ dysfunction, and higher mortality rates [29, 30]. Interestingly, the role of MMP8 varies across different cell types. In a juvenile murine model of polymicrobial sepsis, MMP8 facilitates the clearance of intraperitoneal bacteria, and *Mmp8* deficiency exerts a detrimental effect on the survival outcome in mice with sepsis [31]. This mechanism is associated with the regulatory role of MMP8 in phagocytosis and neutrophil extracellular trap formation [31]. In septic dendritic cells, increased MMP8 expression may be involved in a negative feedback loop, thereby suppressing proinflammatory responses and inducing dendritic cell tolerance in the late phase of polymicrobial sepsis [13]. Our data specifically identify endothelial MMP8 as a driver of sepsis-induced vascular leakage. Above findings suggest that the role of MMP8 is highly context-dependent, varying by developmental age, cell type, and disease stage.

Similar to other MMPs, extracellular MMP8 regulates inflammation by cleaving chemokines as well as other signalling factors [32]. For example, MMP8 can cleave IL-13Rα2 in vitro and in vivo [33]. Recently, research interest in MMPs has shifted to their biological functions in signal transduction. MMP8 can modulate NF-κB p65 nuclear translocation and β-catenin activity in DCs [13]. In the present study, we found that MMP8 could interact with ERK and VE-cadherin and activate ERK signalling to facilitate calpains-mediated VE-cadherin endocytosis in ECs. This observation is in line with a recent report showing that ERK can serve as a stabiliser of the VE-cadherin complex in ECs [17]. However, the exact mechanism by which MMP8 activates ERK remains unclear. Our in vivo data showed that inhibition of MMP8 enzyme activity with M8I in CLP mice suppressed ERK phosphorylation, calpains-mediated endocytosis signalling, and VE-cadherin disruption, suggesting the possible involvement of MMP8 enzyme activity in modulating ERK activation and VE-cadherin disruption. VE-cadherin, a critical component of the adherens junctions, is known to be disrupted during sepsis via mechanisms including VE-cadherin phosphorylation and cleavage by proteases such as MMPs and ADAMs [34]. MMP7, which is increased under septic conditions, is known to be capable of cleaving VE-cadherin [35]. However, the direct cleavage of VE‑cadherin by MMP8 in sepsis has not been clarified.

It is interesting to note that under basal condition *MMP8* overexpression does not obviously alter VE-cadherin protein levels, whereas *MMP8* knockdown markedly increases VE-cadherin levels. This inconsistence could be due to the following facts. VE-cadherin is the molecular basis of gap junctions between ECs, and therefore, is tightly controlled and maintained by intricate and complementary mechanisms. Overexpression of *MMP8* tends to promote the endocytosis of VE-cadherin, impairs gap junctions, and consequently serves as a “danger signal”, whereas *MMP8* knockdown inhibits VE-cadherin disruption, having a weaker deleterious effect. Under basal physiological conditions, the disruption of VE-cadherin induced by *MMP8* overexpression may be rescued by alternative mechanisms, whereas the upregulation of VE-cadherin caused by *MMP8* knockdown poses little threat to cells, which is likely not to be counterbalanced.

This study has some limitations. Firstly, it is worth noting that the in vitro sepsis cell model was established by two different methods: LPS or Cytomix treatment. LPS is the main component of the cell wall of gram-negative bacteria. LPS interacts with the toll-like receptor 4, initiating the immune response by activating many intracellular signalling pathways, particularly the NF-κB signalling pathway [36]. LPS has been widely used for sepsis modelling due to its simplicity, low cost, and strong reproducibility. However, the LPS-induced model only mimics the inflammatory response caused by gram-negative bacterial infection, failing to reproduce the complex pathological microenvironment of clinical sepsis involving multiple pathogens and complex inflammatory responses [37]. In contrast, Cytomix is a cocktail of pro-inflammatory cytokines, typically including TNF-α, IL-1β, and IFN-γ. These three individual cytokine components have long been recognised to be important to the initiation of the septic inflammatory response and resulting ECs and organ injury, and therefore, use of Cytomix is a simplification of the complex septic environment of ECs in vivo, which is comprised of many factors (i.e. bacterial products, activated leukocytes, etc.) that likely drive the increased microvascular permeability [38]. Secondly, although the interaction was identified between MMP8 and ERK, it remains unclear whether it plays a role in ERK activation. Previous studies suggest that MMP8 cleaves the pro-domain of TNF-α and modulates phosphorylation of JNK, ERK, and p38 in microglial cells [39]. TNF-α–dependent MAPK/ERK signalling pathway activation has been reported [40, 41], but the underlying mechanisms remain unclear. The bradykinin B2 receptor (B2R) is a prototypical G protein-coupled receptor that has been shown to activate ERK in endothelial cells [42]. Mukhin et al. demonstrated that MMP8 and MMP13 are involved in B2R-induced EGFR transactivation and subsequent ERK phosphorylation in renal cells in a TGF-α–independent manner [43]. Whether the MMP8-TNF-α-ERK or MMP8-B2R-EGFR-ERK axis functions in ECs in the context of sepsis needs further investigation. Thirdly, we used an MMP8 inhibitor in a mouse model instead of a global or tissue-specific MMP8 knockout mouse model. Previous studies have used MMP8 inhibitors in mouse models to examine sepsis, demonstrating that pharmacological inhibition of MMP8 replicates the phenotype of MMP8 genetic ablation [30, 31]. MMP8 is a secreted protein and can be expressed in a wide range of cells. An endothelial-specific MMP8 knockout mouse model may not provide more accurate results, considering the paracrine effects of MMP8. Finally, the current study enrolled 40 patients with sepsis from our medical centre, providing initial evidence supporting MMP8 as a predictive biomarker for pulmonary vascular leakage in sepsis. The participants were from a single centre, and the sample size was relatively small, which may have influenced the robustness and representativeness of the results. Therefore, the results presented herein should be interpreted with caution, and large-scale multicentre cohorts are required for validation.

In summary, this study provides novel evidence that endothelial MMP8 plays a critical role in promoting pulmonary vascular permeability by inducing VE-cadherin endocytosis during sepsis. We identified that the MMP8-ERK-calpains axis is likely to initiate VE-cadherin internalisation in ECs. We built a nomogram using Alb, BUN, Lac, APACHE II score, and serum MMP8 levels to evaluate the risk of pleural effusion, showing favourable prediction accuracy and good clinical utility. These findings suggest MMP8 as a predictive biomarker and therapeutic target for vascular leakage in sepsis.

## Materials and methods

### Cell culture and treatment

HUVECs were purchased from Cellverse Bioscience (#iCell-h110). HLMVECs were purchased from Aoruicell (#ORCH001A). HUVECs and HLMVECs were authenticated based on immunofluorescence staining for von Willebrand factor (vWF) (Fig. S5a, b). HEK293T and HeLa cell lines were housed in the Laboratory of Oncology at the First Medical Center of Chinese PLA General Hospital. HUVECs were cultured in an iCell Primary Endothelial Progenitor Cell Culture System (Cellverse Bioscience, #PriMed-iCell-002) supplemented with 10% foetal bovine serum (FBS), 1% penicillin/streptomycin solution and 1% EC growth supplement (Cellverse Bioscience, #PriMed-iCell-002a). HLMVECs were cultured in the Primary Microvascular Endothelial Complete Medium (Aoruicell, #ORCCMH001). HEK293T and HeLa cells were cultured in Dulbecco’s modified Eagle medium supplemented with 10% FBS and 1% penicillin/streptomycin. All cells were cultured at 37 °C in a humidified incubator with 5% CO_2_. For the *in vitro* septic EC model, confluent HUVECs or HLMVECs were stimulated with lipopolysaccharide (LPS; 10 μg/mL dissolved in saline; MCE, #HY-D1056) for 12 h or a pro-inflammatory cytokine mixture (Cytomix; IL-1β [10; Proteintech, #HZ-1164], IFN-γ [10; Proteintech, #HZ-1301], and TNF-α [10 ng/mL; Proteintech, #HZ-11024]) for 6 h. Confluent HUVECs or HLMVECs were treated with an ERK inhibitor (LY3214996; 5, dissolved in DMSO; MCE, #HY-101494), a calpain inhibitor (Calpeptin; 10, dissolved in DMSO; MCE, #HY-100223), and a dynamin inhibitor (Dynasore; 20 μΜ, dissolved in DMSO; MCE, #HY-15304) for 1 h followed by septic challenge. To block the lysosomal or proteasomal pathway, confluent HUVECs were treated with chloroquine (100 μΜ for 30 min, dissolved in DMSO; MCE, #HY-17589A) or MG-132 (1 μΜ for 12 h, dissolved in DMSO; MCE, #HY-13259) prior to LPS stimulation.

### RNA-seq and pathway enrichment

LPS- or saline-treated HUVECs and MMP8 overexpressed or control HUVECs were disrupted and homogenised in TRIzol. Library construction and RNA-seq were conducted by Berry Genomics (Beijing, China) as previously described [44]. DEGs were identified using the DESeq2 package in R (R Foundation for Statistical Computing, Vienna, Austria). DEGs between MMP8-overexpressed and control HUVECs were subjected to GO and KEGG analyses for pathway enrichment using the clusterProfiler package in R.

### Haematoxylin and eosin (H&E) staining

Fresh lung tissues were collected and fixed in 4% paraformaldehyde for 48 h, dehydrated, embedded in paraffin, sectioned into 4 µm-thick slices, and stained with H&E as per standard procedures. Sections were observed under an optical microscope, and five non-marginal fields were randomly captured. Two blinded pathologists independently assessed lung injury according to the Smith scoring system [45].

### Mouse model and in vivo treatment of pharmacologic MMP8 inhibitor

C57BL/6 mice were obtained from Beijing Vital River, a joint venture of Charles River Laboratories in China. All the mice were fed under specific pathogen-free conditions at the Laboratory Animal Center of the Chinese PLA General Hospital, where they received a healthy diet and sterile water. The mice were utilised for follow-up experiments at 8 to 10 weeks of age. Polymicrobial sepsis was induced by CLP as described previously [46]. Briefly, mice were anaesthetised with sodium pentobarbital (50 mg/kg i. p.), the abdomen was shaved and the caecum was exposed through a 1-cm midline incision. The caecum was ligated at half the distance between the distal pole and the caecum base using a 4–0 silk suture. Caecal perforation was performed via a single through-and-through puncture midway between the ligation site and tip of the caecum. After needle removal, a small amount of faeces was extruded. Immediately after surgery, a single dose of saline was subcutaneously injected into the resuscitated mice. Sham mice underwent the same procedures without CLP.

To investigate whether MMP8 deficiency in septic mice affects vascular barrier integrity, in vivo treatment with M8I (Santa Cruz, #236,403–25-1) was performed as previously described [31]. Briefly, M8I was dissolved in 1% DMSO in PBS to a final concentration of 0.03 mg/mL. Mice received a 0.3 mg/kg dose of M8I via intraperitoneal injection every 12 h beginning 24 h pre-CLP. Control mice were administered an equivalent volume of 1% DMSO in PBS. Mice were randomly divided into four groups: sham mice (*n* = 5), CLP mice (*n* = 5), CLP mice pretreated with M8I (*n* = 5), and CLP mice pretreated with M8I (*n* = 5). All animal experiments were conducted in accordance with the ethical policies and procedures approved by the Institutional Animal Care and Use Committee of the Chinese PLA General Hospital (approval No. 2023-X19-57).

### BALF harvest

BALF was performed as previously described [47]. Briefly, a catheter was created by inserting a 23-G needle into a transparent plastic polyethylene 21-G tubing before the BALF procedure. 1 mL of sterile saline with 100 µM EDTA was instilled into the mice lungs using the catheter, and BALF was recovered after three lavage procedures. A bicinchoninic acid assay was used to measure the protein concentration in BALF.

### VE-cadherin endocytosis assay

The endocytosis assay was performed mainly as previously described [48]. Overall, 5 × 10^4^ HUVECs were plated on coverslips, allowed to grow for 24 h and then treated with 10 μg/ml LPS or saline for an additional 12 h. To visualise VE-cadherin endocytosis, HUVECs were incubated with anti-VE-cadherin antibody (5 μg/mL; Santa Cruz, #sc-52751) in the presence of 100 µM of the lysosomal inhibitor chloroquine (HY-17589A, MCE) at 37 °C for 4 h. Surface-bound antibodies were removed by exposure to mild acid buffer (2 mM PBS-glycine, pH 2.0) for 10 min and washed twice with PBS. The cells were fixed with 4% paraformaldehyde, incubated with an Alexa Fluor 568-labelled secondary antibody (1:500; Abcam, #ab175701) at 37 °C for 1 h, and detected with an Olympus FV1000 confocal microscope (Tokyo, Japan).

### Patients and samples

Patients with sepsis were recruited from the Emergency Department (ED) of The First Medical Center of Chinese PLA General Hospital between June 2025 and November 2025. Participants were enrolled based on the Sepsis-3 definition criteria, which require known or suspected infection and an acute increase of more than 2 points in the SOFA score [49]. The exclusion criteria were as follows: (1) aged < 18 or > 80 years; (2) sepsis onset exceeding 24 h; (3) diagnosed with malignancies or severe chronic heart or renal diseases prior to admission; (4) pregnant or breastfeeding women. 40 eligible patients comprised the sepsis cohort and 12 age- and sex-matched healthy individuals were recruited as controls.

Venous blood samples (5 mL) were collected within the first 24 h of ED admission. Serum was isolated by centrifugation and stored at −80 °C until use. Serum MMP8 concentration was quantified using a commercial ELISA kit (Elabscience, #E-EL-H145). Clinical parameters—including age, sex, white blood cell count, neutrophil percentage, haemoglobin, Hct, platelet, C-reactive protein, IL-6, procalcitonin, D-dimer, Fib, total protein, Alb, total bilirubin, Cr, BUN, potassium, sodium, Lac, SOFA score and APACHE II score—were recorded. Chest CT was performed to examine pleural effusion. This study was approved by the Ethics Committee of Chinese PLA General Hospital (approval No. S2025-386–01). Written informed consent was obtained from all participants or their legally authorised representatives before enrolment. All procedures involving human participants complied with the principles of the Declaration of Helsinki.

### Statistical analysis

Statistical analyses were performed using GraphPad Prism (version 10.1.2; GraphPad Software, San Diego, CA, USA) and R (version 4.1.2; R Foundation for Statistical Computing). Data are presented as mean ± standard deviation. Statistical differences between two groups were compared using the unpaired Student’s t-test or Mann–Whitney U test, whereas comparisons among multiple groups were conducted using one-way or two-way ANOVA. Survival analysis was performed using Kaplan–Meier curves, and survival differences were analysed using the log-rank test. Correlations between clinical parameters were assessed using Spearman’s correlation analysis. Statistical significance was set at *p* < 0.05.

## Supplementary Information

Supplementary Material 1.

## Data Availability

The raw sequence data reported in this paper have been deposited in the Genome Sequence Archive in National Genomics Data Center, China National Center for Bioinformation/Beijing Institute of Genomics, Chinese Academy of Sciences (GSA-Human: HRA018234) that are publicly accessible at https://ngdc.cncb.ac.cn/gsa-human. Other data supporting the findings of this study are available within the paper and its Supplementary Information.

## Abbreviations

MMPs: Matrix metalloproteinases
ECs: Endothelial cells
HUVECs: Human umbilical vein endothelial cells
CLP: Caecal ligation and puncture
PCR: Polymerase chain reaction
DEGs: Differentially expressed genes
GO: Gene Ontology
KEGG: Kyoto Encyclopedia of Genes and Genomes
OD: Optical density
LPS: Lipopolysaccharide
TEER: Trans-endothelial electrical resistance
M8I: Matrix metalloproteinase 8 inhibitor Ⅰ
EB: Evans blue
AST: Aspartate aminotransferase
BALF: Bronchoalveolar lavage fluid
Co-IP: Co-immunoprecipitation
ED: Emergency department
WBC: Blood cell count
N%: Neutrophil percentage
Hb: Haemoglobin
Hct: Haematocrit
Plt: Platelet
CRP: C-reactive protein
IL-6: Interleukin-6
PCT: Procalcitonin
Fib: Fibrinogen
TP: Total protein
Alb: Albumin
TBil: Total bilirubin
Cr: Creatinine
BUN: Blood urea nitrogen
Lac: Lactate
SOFA: Sequential Organ Failure Assessment
APACHE II: Acute Physiology and Chronic Health Evaluation II
CT: Computed tomography
DCA: Decision curve analysis
B2R: Bradykinin B2 receptor
