# Supplementary material for: MMP8 exacerbates sepsis-induced pulmonary vascular leakage by disruption of endothelial VE-cadherin through ERK signalling
Source: Mol Biomed. 2026 Jul 24;7:118. doi: 10.1186/s43556-026-00522-4 (PMC13400542; doi:10.1186/s43556-026-00522-4)
Supplement: Supplementary file 1 — Supplementary Material 1. [file 43556_2026_522_MOESM1_ESM.docx]

**Title:** **MMP8 exacerbates sepsis-induced pulmonary vascular leakage by disruption of endothelial VE-cadherin** **through ERK signalling**

**Authors:** Yaojun Peng^1, 2†^, Qiyan Wu^3, 4†^, Yuyu Liu^1, 2^, Di Jing^1^, Yang Bai^1^, Haiyan Zhu^1*^

**Authors’ affiliations:**

^1^Department of Emergency, The First Medical Center of Chinese PLA General Hospital, Beijing, China

^2^Medical School of Chinese PLA, Beijing, China

^3^Laboratory of Oncology, The First Medical Center of Chinese PLA General Hospital, Beijing, China

^4^Institute of Oncology, The Fifth Medical Center of Chinese PLA General Hospital, Beijing, China

^†^These authors have contributed equally to this work

^*^Corresponding author: Haiyan Zhu, xiaoyanzibj301@163.com

**Supplementary Material and Methods**

**Plasmids and transfection**

Lentivirus constructs containing the full frame of human *MMP8* cDNA (pCDH-CMV-MMP8-EF1-CopGFP-T2A-Puro) or *MMP8* specific shRNA oligonucleotides (pLent-U6-shRNA-CMV-copGFP-P2A-Puro) were purchased from Vigene Biosciences (Jinan, China). HEK293T cells were co-transfected with lentiviral constructs and packing plasmids to form *MMP8*-overexpressed or knockdown viral particles using Lipofectamine 2000 (Invitrogen, #11668019). Viral supernatants were collected 48 h post-transfection. Stable overexpression or knockdown of *MMP8* in HUVECs was achieved by transduction of viral supernatants in the presence of polybrene (5 μg/mL; Solarbio, #H8761) and subsequent puromycin (5 μg/mL; Solarbio, #IP12803) selection for 48 h. Used shRNA oligonucleotides are listed in **Table S2**.

Expression plasmids containing Flag-tagged *MMP8* (pcDNA3.1-MMP8-C-3×Flag), HA-tagged *ERK1* (pcDNA3.1-ERK1-C-HA) or HA-tagged *ERK2* (pcDNA3.1-ERK2-C-HA) was purchased from GenePharma (Suzhou, China). HeLa cells were transiently transfected with the relevant plasmids using Lipofectamine 2000 (Invitrogen, #11668019).

**Polymerase chain reaction (PCR)**

Total RNA was extracted using TRIzol (Invitrogen, #15596018CN). cDNA was synthesized from 5 μg RNA using a RevertAid RT Reverse Transcription Kit (Thermo Scientific, #K1622). qPCR was performed in triplicate using the StepOnePlus Real-Time PCR System (Applied Biosystems) and GoTaq qPCR Master Mix Kit (Promega, #A6001). Gene expression was normalized to *GAPDH*, and relative expression was quantified using the 2⁻ΔΔCt method. *MMP8* overexpression or knockdown in HUVECs was examined by semi-qPCR using 2×Taq PCR StarMix with Loading Dye (GenStar, #A012). PCR products were visualized using agarose gel electrophoresis. Used primer sets are listed in **Table S3**.

**Immunofluorescence**

Cells and mouse tissues were fixed with 4% paraformaldehyde, permeabilized with 0.2% Triton X-100, and blocked with 10% goat serum. Subsequently, cells and mouse tissues were incubated overnight at 4°C with primary antibodies in darkness, followed by incubation with appropriate fluorophore-conjugated secondary antibodies at room temperature for 1 h. Finally, nuclei were stained with DAPI (Santa Cruz, #sc-359850) at room temperature for 15 min. Images were captured using an Olympus FV1000 confocal microscope (Tokyo, Japan) and processed using the ImageJ software (version 1.8.0, NIH). Used antibodies are listed in **Table S4**.

**Cell viability assay**

A CCK-8 kit (Dojindo, #CK04) was used to analyze cell viability. HUVECs or HLMVECs were seeded in 96-well microplates at a density of 3,000 cells/well and incubated for 12 h to allow attachment. The medium was changed to include LPS or Cytomix. Cells were further cultured for 12 h (for LPS) or 6 h (for Cytomix) before adding CCK-8 reagent. After incubation at 37°C in darkness, the optical density (OD) at 450 nm was measured in each well using a Multiskan FC analyzer (Thermo Scientific).

**Flow cytometry for apoptosis detection**

Apoptosis was detected using Annexin V-APC Apoptosis Detection Kit (KeyGEN BioTECH, #KGA1105). HUVECs were collected via trypsin digestion after treatment with LPS or Cytomix. The cells were washed twice and resuspended in 1×binding buffer containing Annexin V-APC and PI. Cells were incubated in the dark at room temperature for 15 min before detection using flow cytometry (Beckman Coulter). Apoptosis was analyzed using the FlowJo software (Ashland, version 10.8.1).

***In vitro* endothelial permeability assays**

HUVECs were seeded in 24-well transwell inserts (0.4-μm pore diameter; Corning, #CLS3413) at a density of 5 × 10^4^ cells per well and cultured 24 h to form an intact endothelial monolayer. The culture medium was replaced with LPS or Cytomix-containing medium. Endothelial permeability was assessed at 0, 3 and 6 h post-treatment by measuring TEER using an EVOM2 volt ohmmeter (World Precision Instruments). The FITC-dextran flux was measured at the end of the TEER analysis. The culture medium of upper chamber was replaced with 250 μg FITC-dextran (70000 MW; MCE, #HY-128868E) containing medium (200 μL). One hour later, 100 μL of the lower chamber medium was transferred to a 96-well plate, and fluorescence intensity at excitation/emission wavelength of 485/535 nm was measured using a SpectraMax M2 fluorescence microplate reader (Molecular Devices).

***In vivo* pulmonary vascular permeability assay**

To evaluate pulmonary vascular permeability *in vivo*, mice were pretreated with M8I pre-CLP to induce sepsis. At 20 h post-CLP, the mice were intravenously injected with 0.5% EB dye (40 µg/g, dissolved in PBS; MCE, #HY-B1102) via the tail vein. After 1 h, mice were anaesthetized with sodium pentobarbital (50 mg/kg, i.p.). A clean hypodermic needle was inserted into the right ventricle for perfusion and the left atrium was incised for outflow. Next, 10–15 mL of ice-cold PBS containing 2 mM EDTA was gently injected into the right ventricle until the effluent was clear. The lungs were excised, weighed and imaged. To extract extravascular EB dye, the lungs were incubated in 500 µL of formamide at 55°C for 48 h. After incubation, 100 µL of supernatant was collected, and the OD at 620 nm was measured using the Multiskan FC analyzer. In parallel, a standard curve was generated by serial dilution of EB in formamide, and the total extravascular EB was calculated based on this and normalized to lung weight.

**Biochemical analysis**

The mice were anaesthetized before blood samples were collected via heart puncture. The blood samples were centrifuged at 3700 rpm for 20 min at room temperature, and serum was stored at -20°C until use. A mouse NT-proBNP ELISA kit (Elabscience, #E-EL-M0834), Cr colorimetric assay kit (Elabscience, #E-BC-K188-M), and AST activity assay kit (Elabscience, #E-BC-K236-M) were used for heart, renal, and liver function tests, respectively.

**Measurement of MMP8 activity**

MMP8 activity in mice serum samples was determined using a commercially available fluorimetric kit (Anaspec, #AS-71133) according to the manufacturer’s instructions. The kit can detect MMP8 activity by examining the fluorescence released from the fluorescence resonance energy transfer (FRET) peptide after cleavage by MMP8. The fluorescence intensity can be monitored at excitation/emission wavelength of 340/490 nm using a SpectraMax M2 fluorescence microplate reader (Molecular Devices).

**Molecular docking**

The AlphaFold software (https://alphafoldserver.com/) was used to predict rigid docking between MMP8 and ERK1 or MMP8 and ERK2. We searched the UniProt database (https://www.uniprot.org/) for MMP8 (UniProt identifier: P22894), ERK1 (UniProt identifier: P27361) and ERK2 (UniProt identifier: P28482) and sourced to the AlphaFold Protein Structure Database (https://alphafold.ebi.ac.uk/) to acquire the protein sequences (MMP8 [AF-P22894-F1-v6], ERK1 [AF-P27361-F1-v6], ERK2 [AF-P28482-F1-v6]). These sequences were uploaded onto the AlphaFold server. Complex structure prediction was performed according to the standard pipeline of AlphaFold. The top-ranked complex structural model was selected and energy minimization was performed using the AMBER22 tool (https://ambermd.org/) by applying an ff14SB force field. The energy-minimized model was evaluated for binding energy using the Prodigy tool (https://wenmr.science.uu.nl/prodigy/). Molecular graphic visualization was performed using PyMol (http://www.pymol.org).

**Co-Immunoprecipitation (Co-IP)**

Co-IP was performed using an agarose-based Pierce Co-IP Kit (Thermo Scientific, #26149), which enables isolation of native protein complexes from a lysate or other complex mixture by directly immobilizing the purified antibodies onto an agarose support. Briefly, expression plasmids, including Flag-tagged MMP8, HA-tagged ERK1 and HA-tagged ERK2, were transiently transfected into HeLa cells using Lipofectamine 2000 (Invitrogen, #11668019). 48 h post-transfection, the cells were lysed, and protein concentrations were measured. Then, 500–1000 µg of protein was pre-cleared by the control agarose resin, and added to a column containing 5 µg of anti-HA antibody (Invitrogen, #26183) or anti-Flag antibody (Invitrogen, #701629) immobilized resin. After overnight incubation at 4℃ on a rotator, non-bound proteins were removed by washing. The precipitated proteins were harvested by elution from the resin. The lane marker buffer was added to the precipitated proteins, and heated at 100°C for 5 min before applying to Western blotting. Used antibodies are listed in **Table S4**.

**Western blotting**

The cells and mouse tissue samples were homogenized in RIPA lysis buffer containing 1 mM phenylmethylsulfonyl fluoride (Solarbio, #P0100), protease inhibitors (GenStar, #E128) and phosphatase inhibitors (Solarbio, #IKM1060). The lysates were quantified using BCA assay. Then, 20–30 µg of total protein was separated via sodium-dodecyl-sulphate-polyacrylamide gel electrophoresis and transferred onto a polyvinylidene fluoride membrane (Millipore, #IPVH00010). Membranes were treated with 5% skimmed milk for 1 h at room temperature to block nonspecific binding. Blots were incubated with primary antibodies at 4°C overnight, then probed with appropriate horseradish peroxidase (HRP)-conjugated secondary antibody for 1 h at room temperature. HRP-based detection was performed using a chemiluminescence imaging system (Tanon Life Science). The antibodies used are listed in **Table S4**.

**Nomogram for predicting** **sepsis-induced pulmonary vascular leakage**

A nomogram was constructed to calculate the risk of sepsis-induced pulmonary vascular leakage. A sepsis cohort (n = 40) was used to train the model. Affliction with pleural effusion in patients with sepsis was examined using chest CT, and applied as the dependent variable. Univariate logistic regression and a machine learning method—namely, SVM-RFE—were employed to identify candidate features from multiple clinical parameters. Univariate logistic regression was implemented using the glmnet package in R, with p < 0.1 as the screening criterion. In parallel, SVM-RFE was performed using the e1071 package in R with a 10-fold cross-validation to rank features based on their contribution to disease classification. Intersection analysis was performed to identify the common candidate features screened using the above two methods. The pROC package was used to conduct ROC curve analysis. Nomogram construction, calibration curve analysis, and DCA were conducted using the RMS package.

**Supplementary Figures**

**
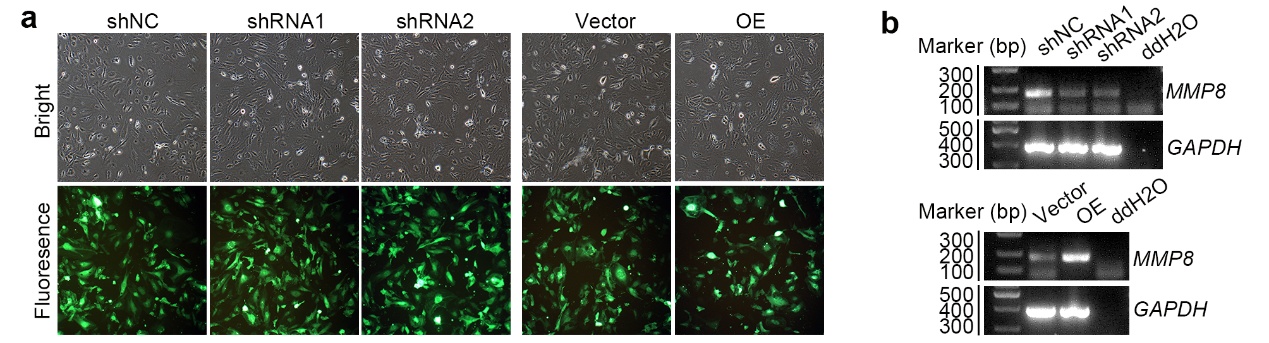
**

**Fig. S1** Knockdown or overexpression of *MMP8* in HUVECs by lentiviral transduction. **a** Gene transfer efficiency was examined by GFP fluorescence. **b** Expression of *MMP8* assessed by PCR amplification followed by agarose gel electrophoresis.

**
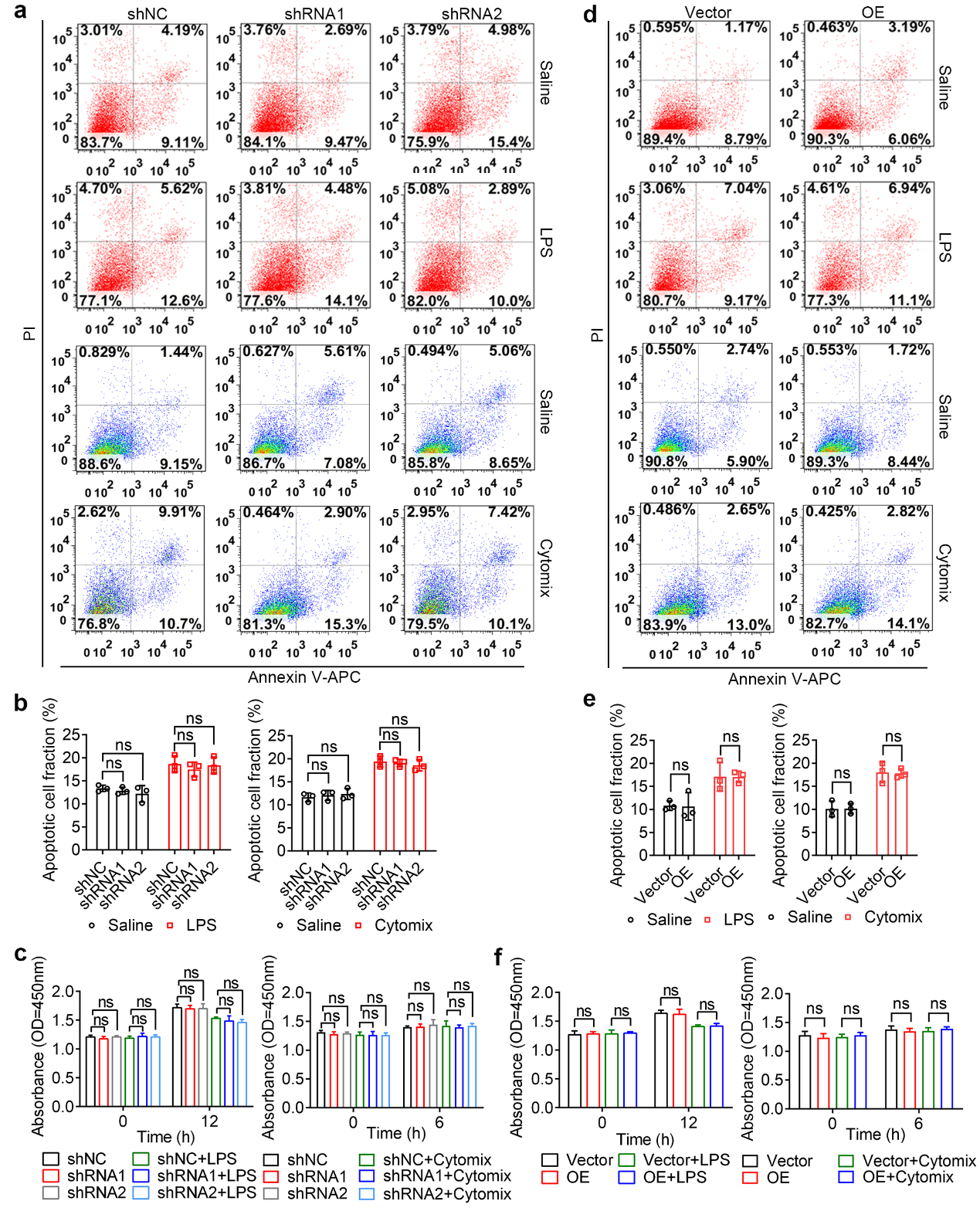
**

**Fig. S2** Effect of MMP8 on apoptosis or viability of HUVECs. **a** Representative Annexin V‑PI dot plots for apoptosis detection in *MMP8*-knockdown HUVECs or control cells and its quantification **(b)** (n = 3 per group). **c** Bar plots showing cell viability assessed by CCK8 method in *MMP8*-knockdown HUVECs or control cells (n = 3 per group). **d** Representative Annexin V‑PI dot plots for apoptosis detection in *MMP8*-overexpressed HUVECs or control cells and its quantification **(e)** (n = 3 per group). **f** Bar plots showing cell viability assessed by CCK8 method in *MMP8*-overexpressed HUVECs or control cells (n = 3 per group). Data are shown as mean ± SD; p values were calculated using one-way ANOVA with Donnett’s t test **(b, c**) or unpaired Student’s t test (**e, f**); ns, no significance.

**
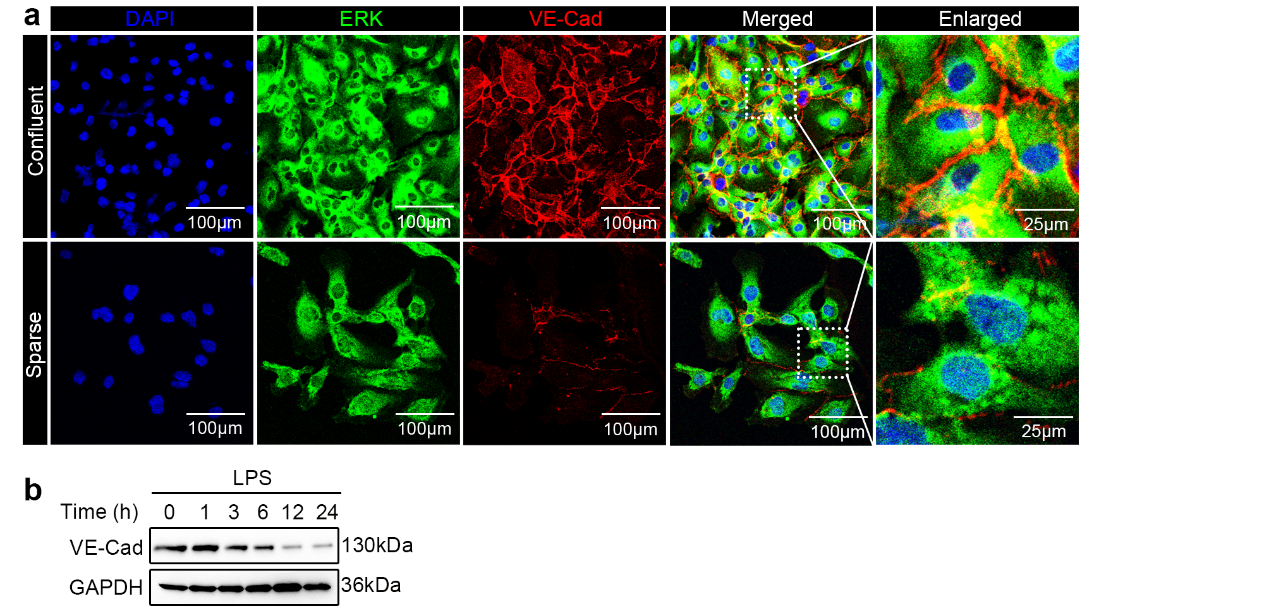
**

**Fig. S3** Expression of VE-cadherin and its colocalization with ERK in HUVECs. **a** Representative immunofluorescence images showing colocalization of VE-cadherin (red) and ERK (green) in HUVECs with different confluence; scale bar: 100 μm for the left four columns and 25 μm for the right column. **b** Representative immunoblots showing time-dependent expression of VE-cadherin in HUVECs upon LPS treatment. VE-Cad, VE-cadherin

**
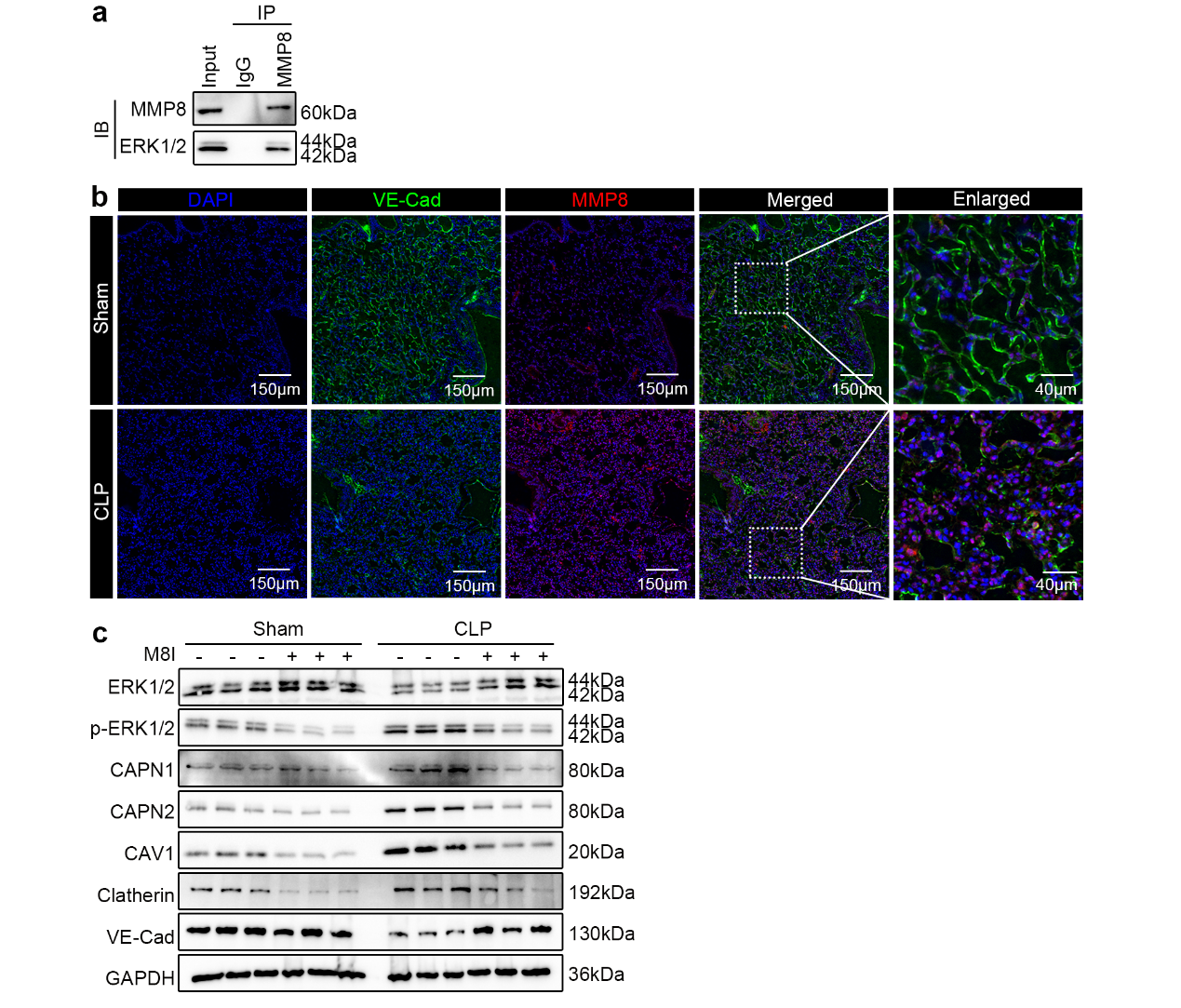
**

**Fig. S4** MMP8 interacts with ERK to promote VE-cadherin disruption in mice lung tissues. **a** Representative immunoblots of Co-IP showing MMP8 and ERK binding in mice lung tissues. **b** Representative immunofluorescence images showing MMP8 (red) and VE-cadherin (green) colocalization in mice lung tissues; scale bar: 150 μm for the left four columns and 40 μm for the right column. **c** Representative immunoblots showing ERK1/2, p-ERK1/2, CAPN1, CAPN2, CAV1, clathrin, and VE-cadherin in the lungs of mice treated with MMP8 inhibitor (M8I) or vehicle. VE-Cad, VE-cadherin


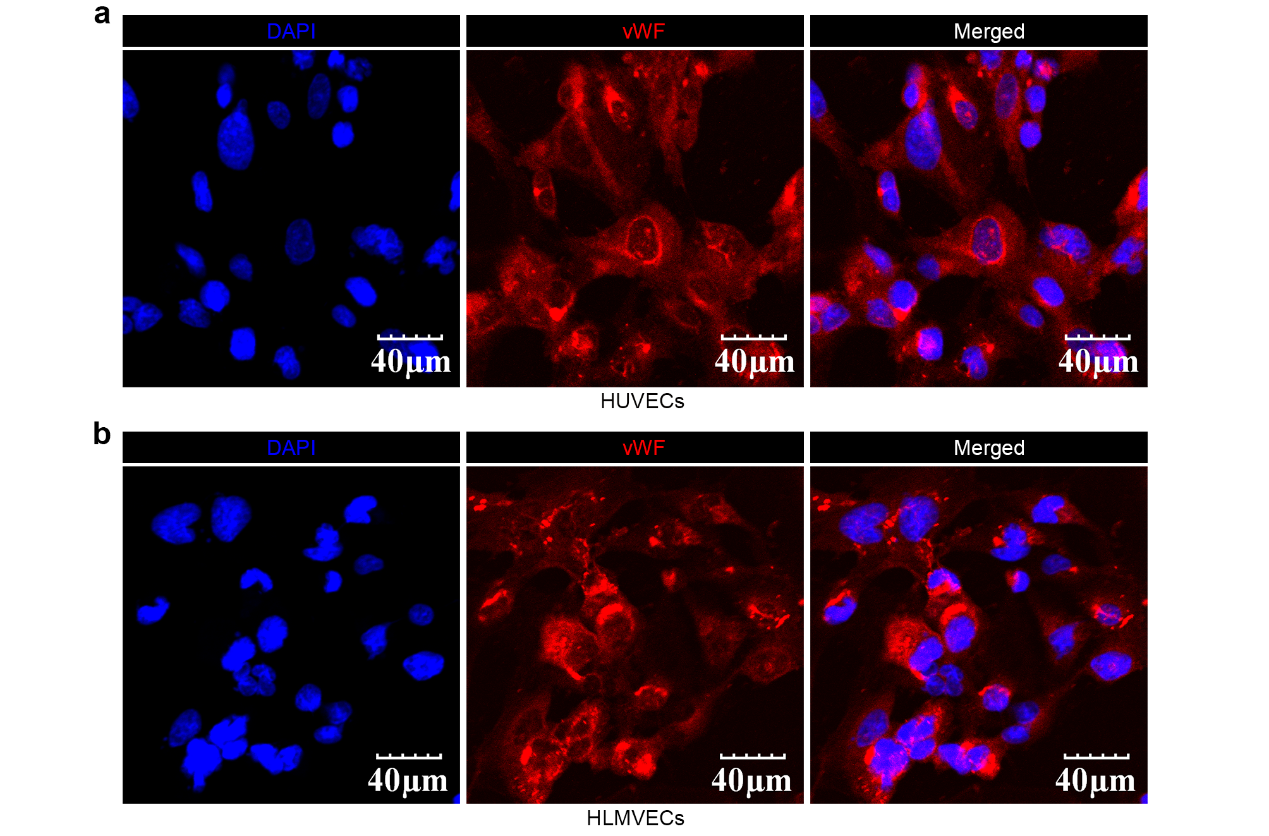


**Fig. S5** Immunofluorescence staining of vWF for HUVECs and HLMVECs authentication. Expression of vWF (red) in HUVECs **(a)** and HLMVEs **(b)**. Scale bar: 40 μm.

**Supplementary Tables**

**Table S1.** Demographic, clinical and laboratory characteristics of participants.

| **Variable** | **Healthy individuals**  **(n = 12)** | **Septic patients** | | **p1** | **p2** |
| --- | --- | --- | --- | --- | --- |
|  |  | **Pleural effusion negative (n = 25)** | **Pleural effusion positive (n = 15)** |  |  |
| **Age** (year) | 56.67 (13.43) | 59.60 (12.71) | 65.27 (13.06) | 0.246 | 0.185 |
| **Sex** (male [%]) | 6 (50) | 12 (48.00) | 6 (40.00) | 0.999 | 0.125 |
| **MMP8** (ng/mL) | 8.198 (4.674) | 35.76 (41.20) | 85.11(79.52) | **0.014*** | **0.014*** |
| **WBC** (×10^9^/L) | - | 14.44 (5.282) | 14.57 (9.163) | - | 0.955 |
| **N%** | - | 90.87 (5.722) | 87.65 (7.012) | - | 0.122 |
| **Hb** (g/L) | - | 123.9 (25.50) | 115.5 (30.06) | - | 0.172 |
| **Hct** (%) | - | 35.78 (7.414) | 33.17 (8.961) | - | 0.324 |
| **Plt** (×10^9^/L) | - | 155.8 (105.3) | 203.0 (140.2) | - | 0.234 |
| **CRP** (mg/dL) | - | 12.87 (12.11) | 12.73 (11.17) | - | 0.972 |
| **IL-6** (pg/mL) | - | 1735 (2108) | 1462 (1946) | - | 0.686 |
| **PCT** (ng/mL) | - | 30.93 (33.46) | 24.77 (38.34) | - | 0.596 |
| **D-dimer** (μg/mL) | - | 6.425 (6.078) | 8.452 (6.554) | - | 0.328 |
| **Fib** (g/L) | - | 4.996 (2.552) | 4.652 (3.462) | - | 0.721 |
| **TP** (g/L) | - | 63.97 (10.65) | 57.47 (9.277) | - | 0.058 |
| **Alb** (g/L) | - | 34.81 (6.377) | 29.57 (3.926) | - | **0.007**** |
| **TBil** (μmol/L) | - | 54.88 (72.89) | 44.99 (80.10) | - | 0.691 |
| **Cr** (μmol/L) | - | 140.0 (112.9) | 199.4 (123.6) | - | 0.128 |
| **BUN** (mmol/L) | - | 11.07 (10.03) | 17.32 (7.040) | - | **0.041*** |
| **K** (mmol/L) | - | 4.141 (1.147) | 4.303 (0.7783) | - | 0.633 |
| **Na** (mmol/L) | - | 136.4 (5.638) | 136.6 (9.146) | - | 0.944 |
| **Lac** (mmol/L) | - | 2.732 (2.193) | 4.141 (1.147) | - | **0.047*** |
| **SOFA score** | - | 5.560 (3.959) | 6.200 (3.342) | - | 0.372 |
| **APACHE II Score** | - | 17.87 (5.805) | 12.60 (8.436) | - | **0.013*** |

Data are shown as mean (SD) or n (%); p1 reports the statistical difference between the healthy individuals and septic patients; p2 reports the statistical difference between the septic patients with and without pleural effusion; p values were calculated by Student’s t test (age, MMP8, WBC, N%, Hb, Hct, Plt, CRP, IL-6, PCT, D-dimer, Fib, TP, Alb, TBil, Cr, BUN, K, Na, and Lac), Mann-Whiteny U test (SOFA score and APACHE II Score), or Fisher’s exact test (Sex); ∗p < 0.05 and ∗∗p < 0.01. WBC: blood cell count; N%: neutrophil percentage; Hb: hemoglobin; Hct: hematocrit; Plt: platelet; CRP: C-reactive protein; IL-6: interleukin-6; PCT: procalcitonin; Fib: fibrinogen; TP: total protein; Alb: albumin; TBil: total bilirubin; Cr: creatinine; BUN: blood urea nitrogen; Lac: lactate; SOFA: Sequential Organ Failure Assessment; APACHE II: Acute Physiology and Chronic Health Evaluation II.

**Table S2.** MMP8 specific and control shRNA oligonucleotides used in this study.

| **Name** | **Sequence (5’-3’)** |
| --- | --- |
| shNC | TTCTCCGAACGTGTCACGTTTCAAGAGAACGTGACACGTTCGGAGAATTTTTT |
| shRNA1 | GCTGAGGTAGAAAGAGCTATCTTCAAGAGAGATAGCTCTTTCTACCTCAGCTTTTTT |
| shRNA2 | GCCATCTATGGACTTTCAAGCTTCAAGAGAGCTTGAAAGTCCATAGATGGCTTTTTT |

**Table S3.** Primer sets used for PCR analysis in this study.

| **Primer name** | **Sequence (5’-3’)** |
| --- | --- |
| *GAPDH*-F | GAGAGAAACCCGGGAGGCTA |
| *GAPDH*-R | GACTCCACGACGTACTCAGC |
| *MMP8*-F | AGCCAGGAGGGGTAGAGTTT |
| *MMP8*-R | TGCATCAGTGCAGTTCCTCT |
| *MMP10*-F | AGTTTGGCTCATGCCTACCC |
| *MMP10*-R | AGTTTGGCTCATGCCTACCC |
| *MMP11*-F | CGACAGAAGAGGTTCGTGCT |
| *MMP11*-R | CCCCGATAGTCCAGGTCTCA |
| *MMP25*-F | GGACTGGCTGACTCGCTATG |
| *MMP25*-R | CCATGTCAGGGTTCGCTTCT |
| *CDH5*-R | CCCACAGGCACGATCTGTT |
| *CDH5*-R | CATTCTTGCGACTCACGCTT |

**Table S4**. Antibodies used in this study.

| **Name** | **Supplier** | **Cat. No.** | **Dilution** |
| --- | --- | --- | --- |
| Rabbit anti-MMP8 pAb | Absin | abs115874 | 1:1000 (WB), 1:250 (IF), |
| Rabbit anti-MMP8 mAb | Absin | abs178140 | 1:100 (Co-IP) |
| Rabbit anti-vWF pAb | Proteintech | 27186-1-AP | 1:250 (IF) |
| Mouse anti-VE-cadherin mAb | Santa Cruz | sc-9989 | 1:500 (WB), 1:250 (IF), 1:50 (Co-IP) |
| Mouse anti-VE-cadherin mAb | Santa Cruz | sc-52751 | 1: 40 (internalisation assay) |
| Rat anti-PECAM-1 (CD31) mAb | Santa Cruz | sc-101454 | 1:250 (IF) |
| Mouse anti-Caveolin 1 mAb | Zenbio | 660110 | 1:1000 (WB) |
| Rabbit anti-Calpain 1 mAb | Zenbio | R381868 | 1:1000 (WB) |
| Rabbit anti-Calpain 2 mAb | Zenbio | R381992 | 1:1000 (WB) |
| Rabbit anti-JNK1/2 mAb | Zenbio | R22866 | 1:1000 (WB) |
| Rabbit anti-phos-JNK1/2 pAb | Zenbio | 381100 | 1:1000 (WB) |
| Mouse anti-p38 pAb | Zenbio | 200782 | 1:1000 (WB) |
| Rabbit anti-phos-p38 pAb | Zenbio | 310091 | 1:1000 (WB) |
| Rabbit anti-ERK1/2 mAb | Zenbio | R22685 | 1:1000 (WB), 1:500 (IF), 1:100 (Co-IP) |
| Rabbit anti-phos-ERK1/2 mAb | Zenbio | R24245 | 1:1000 (WB) |
| Rabbit anti-Clatherin heavy chain mAb | Zenbio | R381847 | 1:1000 (WB) |
| Rabbit anti-GAPDH mAb | Zenbio | R24402 | 1:2000 (WB) |
| Mouse anti-HA mAb | Invitrogen | 26183 | 1:1000 (WB), 1:250 (IF), 1:100 (Co-IP) |
| Rabbit anti-Flag mAb | Invitrogen | 701629 | 1:1000 (WB), 1:250 (IF), 1:100 (Co-IP) |
| Mouse IgG isotype control | Invitrogen | 02-6502 | - |
| Rabbit IgG isotype control | Invitrogen | 02-6102 | - |
| HRP-conjugated goat anti-Rabbit IgG (H+L) | Abbkine | A21020 | 1:5000 (WB) |
| HRP-conjugated goat anti-Mouse IgG (H+L) | Abbkine | A21010 | 1:5000 (WB) |
| Alexa Fluor® 568 preadsorbed goat anti-Mouse IgG H&L | Abcam | ab175701 | 1:500 |
| Alexa Fluor® 647 preadsorbed goat anti-Rabbit IgG H&L | Abcam | ab150083 | 1:500 |
| Alexa Fluor® 647 preadsorbed goat anti-Rat IgG H&L | Abcam | ab150167 | 1:500 |

WB: Western blotting; IF: immunofluorescence; Co-IP: Co-Immunoprecipitation.
